# HERC3 directly targets RPL23A for ubiquitination degradation and further regulates Colorectal Cancer proliferation and the cell cycle

**DOI:** 10.7150/ijbs.72014

**Published:** 2022-05-01

**Authors:** Zhiyuan Zhang, Qi Wu, Meimiao Fang, Yu Liu, Ji Jiang, Qingyang Feng, Ronggui Hu, Jianmin Xu

**Affiliations:** 1 Department of General Surgery, Zhongshan Hospital, Fudan University, 200030 Shanghai, China.; 2 State Key Laboratory of Molecular Biology, Shanghai Institute of Biochemistry and Cell Biology, Center for Excellence in Molecular Cell Science, Chinese Academy of Sciences, 200030 Shanghai, China.; 3 College of Life Sciences, Shanghai Normal University, Shanghai 200234, China.

**Keywords:** colorectal cancer, cell cycle, p21, ubiquitin

## Abstract

**Aims**: Colorectal cancer (CRC) has high mortality and morbidity rates; however, the mechanism of CRC cells uncontrolled proliferation is unclarified, E3 ligases are widely reported to have crucial functions in cancers. HERC3 was once recognized as an important role in CRC, however its effects on CRC cell proliferation and cell cycle are blank.

**Methods**: Correlation between HERC3 and clinical characteristics was analyzed. Coimmunoprecipitation, mass spectrometry analysis and GST-pull down were performed to identify interacting-proteins of HERC3. Expression pattern of RPL23A and its correlation between HERC3 was researched via qRT-PCR, western blot and immunohistochemistry. In vivo and vitro gain-and loss-of-function assays and rescue experiments concentrating HERC3-RPL23A axis in terms of cell proliferation and cell cycle were conducted. The ubiquitination regulatory mechanism between HERC3 and RPL23A were identified via vivo ubiquitylation assays, cycloheximide analysis and mass spectrometry analysis. GSEA aided to research the potential functional mechanism of RPL23A and validated by western blot and in vivo ubiquitylation assays.

**Results**: HERC3 expression decreased gradually from colorectal tissues in healthy individuals to adjacent-tumors normal tissue in CRC patients, and to tumor tissues and HERC3 could inhibit CRC cell proliferation and arrest cells in the G0-G1 phase. RPL23A which was recognized as one potential target of HERC3 was identified to be overexpressed in CRC and could serve as a prognostic biomarker in CRC. RPL23A could also independently regulate the cell cycle and cell proliferation and attenuate the influence of HERC3 on CRC. In addition, HERC3 directly interacted with RPL23A and served as an E3 ligase to ubiquitination degrade RPL23A via K48-dependant manner through the HECT domain. Furthermore, HERC3 could regulate the ubiquitination of p21 and further modulate protein expression of c-Myc and p21 via regulating RPL23A.

**Conclusion**: HERC3 controlled CRC proliferation, the cell cycle and regulated the c-Myc/p21 axis via directly targeting RPL23A for ubiquitination degradation.

## Introduction

Colorectal cancer (CRC) is the most common gastrointestinal tumor worldwide, and its mortality and incidence are on the rise worldwide. According to the latest data, CRC is estimated to rank as the third most common cause of cancer-associated death in the male or female populations respectively and the second in the whole population 1. However, the latent mechanism of the initiation and progression of CRC remains unclarified. Due to the high mortality and morbidity rate of CRC, it is imperative to reveal the developmental mechanism underlying CRC.

The ubiquitin-proteasome system (UPS) has been broadly reported as an important regulator among various cancers. The UPS can be characterized as a cascade that transfers ubiquitin to targeted proteins 2. The UPS primarily consists of three kinds of enzymes to work collectively in a sequential reaction to transmit ubiquitin (Ub) to the downstream substrate. These 3 kinds of enzymes are summarized into Ub-activating enzymes, Ub-conjugating enzymes, and E3s (Ub-protein ligases) 3. Notably, E3 ligases are widely recognized as the important components of the UPS that recruit ubiquitin-loaded E2s and recognize downstream substrates; moreover, the specificity of the degraded substrate is usually dependent on the E3 ligase 4. Due to the structure and functional mechanism, E3 ligases are commonly categorized into 2 principal families, including the homologous to the E6-AP c terminus (HECT) and really interesting new gene (RING) finger families 4-6. Growing evidence indicates that aberrantly expressed E3 ubiquitin ligases may exert significant functions in the development of cancer and can also influence the response to treatment, indicating that E3 ligases could be druggable and may help to explain the latent mechanism of the progression of cancer.

In our previous work, we used 2 steps differential analysis to identify the key E3 in CRC 7. However, it cannot reflect the initiation and progression process of CRC which disturbs normal individual to cancer patients. To avoid the drawbacks, we modified the differential analysis as 3 steps differential analysis. We screened out the genes that showed the same aberrant expression trend from colorectal tissues in healthy individuals to adjacent-tumor normal tissue in CRC patients, and to tumor tissues. Taken with the clinical information together, interestingly, HERC3 was enriched once again indicating its crucial role in colorectal cancer.

HERC3 belongs to the HERC E3 family, which has HECT and RCC1-like domains and owns six family members. These six members are usually divided into two subgroups according to the size of the proteins. Many researchers previously reported that HERC E3s have significant functions in various cancers. HERC1 used to be reported to influence the metastasis of breast cancer 8. HERC2 was indicated to own a critical function in the regulation of the p53/MDM2 axis 9. HERC4 could induce breast cancer progression by downregulating the tumor suppressor LATS1^10^. Bioinformatic analysis indicated that HERC5 could serve as a prognostic biomarker in breast cancer 11.

In this study, we identified that HERC3 was gradually reduced from colorectal tissues in healthy individuals to adjacent-tumors normal tissue in CRC patients, and to tumor tissues. We previously identified HERC3 to inhibit the metastasis via ubiquitination degradation EIF5A2 in CRC 7. Nevertheless, the most important characteristics of tumorigenesis in normal individuals is still the uncontrolled cell proliferation, and the functions of HERC3 in CRC cell proliferation is still blank. Here, we discovered for the first time that HERC3 was associated with the tumor size, suppressed CRC cell growth and blocked the cell cycle at the G0-G1 phase. Besides, we previously discovered that EIF5A2 had almost non effect in regulating CRC cell proliferation and cell cycle indicating that HERC3 could control cell proliferation and cell cycle via other targets 7. In this work, RPL23A was the first time to identified as the direct target of HERC3 to be responsible for the effects of HERC3 on cell cycle and cell proliferation. Moreover, RPL23A was also the first time to be identified as an oncogene that was overexpressed and could function as a prognostic index for CRC. In addition, RPL23A could independently regulate the cell cycle and cell proliferation of CRC, and further impair the effect of HERC3 on CRC cells, indicating that HERC3 exerted its functions via RPL23A. Mechanistically, HERC3 could directly bind and degrade RPL23A via the ubiquitination proteasome-dependent pathway. We additionally proved that HERC3 could modulate the ubiquitination of p21, and regulate the expression of c-Myc and p21, besides, RPL23A rescued the effects of HERC3 on ubiquitination of p21 and also the effects on protein expression of p21 and c-Myc. In summary, to our knowledge, this is the first report demonstrating that HERC3 controls CRC proliferation and the cell cycle via directly targeting RPL23A for ubiquitination degradation. Our discovery may provide novel research directions into the study and treatment of CRC.

## Materials and Methods

### Obtain and process of the raw data

The raw data were acquired from the XENA website (http://xena.ucsc.edu/). The raw microarray data mainly included two types: the expression matrix of normal colorectal epithelial tissues was obtained from the Genotype-Tissue Expression (GTEx) database and the expression matrix of CRC samples and corresponding normal samples were achieved from The Cancer Genome Atlas (TCGA) database. The two data sets were combined and normalized according to the respective descriptions of the two databases from the XENA website. Differential analysis was performed with Wilcoxon test. Without any special criterion for fold change plus False discovery rate (FDR) < 0.05 were set as the selection threshold. The gene list of E3 ligases was obtained from the research conducted by Ge *et al*. 12. OS and DFS analysis were conducted by GEPIA2 (http://gepia2.cancer-pku.cn/#index). The differentially expressed E3s were identified through 3-times differential expression analysis: First, differentially expressed E3 ligases were identified between healthy colorectal tissues in GTEx and tumor-adjacent normal tissues in the TCGA; second, differentially expressed E3 ligases were identified by comparing tumor-adjacent tissues and tumor tissues in CRC patients from TCGA; third, healthy colorectal tissues in GTEx were combined with tumor-adjacent tissues in TCGA and further compared with CRC tissues in the TCGA to identify the differentially expressed E3 ligases. The genes that shared the same differential expression trend (upregulated or downregulated) were identified by intersecting the 3-times differentially analysis results.

### Patient specimens

All patients signed informed consent. 50 CRC samples and relevant matched tumor-adjacent samples that were used to detect the mRNA and protein expression of corresponding genes or proteins were acquired from patients who received CRC radical surgeries in 2019. These samples were utilized to detect the expression feature of relevant genes or proteins in tumors and paired CRC patients' adjacent normal tissues through qRT-PCR, IHC and western blotting. A total of 200 CRC tumor samples from CRC patients who received radical surgeries from 2008-2012 were also included in this study to evaluate the association between the corresponding proteins expression and clinical characteristics by IHC. Samples enrolled were all obtained from Zhongshan Hospital Fudan University, and no patients received treatment before the radical surgeries. The study was ethically approved by the corresponding ethics committee.

### Cell culture and reagents

Cells including DLD-1, HCT116, SW480, SW620, and HEK293T were acquired ATCC, besides, NCM460 was acquired from INCELL. All these cells were cultured under DMEM (Corning) plus with 10% FBS and penicillin/streptomycin. All cells were cultured in an incubator at 37°C under 5% CO_2_.

### Construction and transfection of plasmids

The plasmids including pcDNA3.0-RPL23A-Flag and relevant point mutants, pcDNA3.0-p21-Flag, pcDNA3.0-RPL23A-Myc, pcDNA3.0-RPL23A-6His, pET22b-RPL23A-6His, and pET28A-His-RPL23A were built in our laboratory. Construction of some other plasmids and transfection of the plasmids are available in our previous published work 7.

### Construction of stable cell lines

Overexpression lentivirus targeting HERC3 and RPL23A were built in GeneChem, Shanghai based on the relevant NCBI reference (reference of HERC3: NM_014606, reference of RPL23A: NM_000984), shHERC3 shRPL23A lentivirus and relevant control lentivirus were also constructed in GeneChem, Shanghai, and cells were infected following the instructions. The shRNA sequences of HERC3 and control could be found in our previous work 7. The shRNA sequences of RPL23A were set as: shRPL23A: 5'-GAAGCTGTATGACATTGAT-3'.

### Antibodies

anti-His (66005-1-Ig), Anti-c-Myc (10828-1-AP), anti-Flag (20543-1-AP), anti-p21 (10355-1-AP), anti-GAPDH (60004-1-Ig), and anti-GST (HRP-66001) were acquired from Proteintech, anti-Ki67 (ab92742) was obtained from abcam. Anti-HERC3 (HPA039170,), anti-HA (SAB4300603), anti-RPL23A (HPA063979, dilution 1:500), and anti-Myc (SAB4301136) were acquired from Sigma. The antibodies were utilized following the manual.

### qRT-PCR

Total RNA Kit (Tiangen, China) was utilized to obtain total RNA from patient samples and CRC cells treated with TRIzol (Invitrogen) in advance. ReverTra Ace qPCR RT Master Mix (Toyobo, Japan) was utilized to reverse transcription of total RNA into complementary DNA (cDNA). Real-time PCR instrument (Thermo Fisher, ABI7500) was used to conduct the qRT-PCR, and the expression of the target transcript was normalized to that of GAPDH through the 2^ΔΔCT^ method. The primers were as follows: HERC3 forward: 5'-CTCTGGCAGATCAGCATATCATT-3', HERC3 reverse: 5'-CAGCTTTTGTATTAACCTGGGCA-3', GAPDH forward: 5'-GGAGCGAGATCCCTCCAAAAT-3', and GAPDH reverse: 5'-GGCTGTTGTCATACTTCTCATGG-3'.

### Immunoprecipitation (IP), western blot, coimmunoprecipitation (co-IP), IHC and immunofluorescence

More details were shown in our previous published paper 7.

### Exploration of HERC3-interacting proteins by mass spectrometry and prediction of the potential interaction sites

HERC3-Flag plasmids were utilized to transfect into HCT116 cells, cells were then harvested after 48 hours. Anti- Flag affinity gels were used to enrich the cells, and 8 M urea buffer was used to further eluted samples. The samples were then subjected to mass spectrometry analysis.

For prediction of the detailed interaction sites between HERC3 and RPL23A, methods could be found in our previous work 7.

### Purification of the corresponding recombinant proteins

BL21 *Escherichia coli* cells were used to express the relevant proteins with the indicated tags (GST or His-tags). Then, isopropyl β-D-thiogalactoside (IPTG, Sigma-Aldrich) was utilized to induce, pellet, lysed cells and samples were further incubated together with Ni^2+^ nitrilotriacetic acid (NTA) beads. The products were washed with 20 mM glutathione (GSH, Sigma). More details were available in our previous published work 7.

### GST pulldown

Glutathione sepharose 4B compounded with purified relevant GST-tagged proteins (20 μg) or His-tagged proteins (20 μg) then were incubated with 500 μl pulldown buffer (100 mM NaCl, 20 mM Tris-Cl, 5 mM MgCl_2_, 1 mM DTT, 0.5% NP-40, 1 mM EDTA, and 10 μg/ml BSA pH 7.5). The beads were washed by pulldown buffer for five times. Afterwards, the beads were denatured and then processed for Western blotting.

### Cycloheximide analysis and in vivo ubiquitylation assay

Corresponding plasmids were used to transfect cells. MG-132 was used to treat the cells before harvesting. Cells were lysed, and IP was performed with Flag affinity gels as previously described. After incubation, the Flag affinity gels were then washed for 5 times by RIPA buffer and denatured with indicated materials. Western blotting was then carried out. Cycloheximide analysis: CHX (100 µg/ml) was used to treat the cells and samples were obtained at the indicated set times for western blotting.

### Ubiquitination modification sites identified by mass spectrometry analysis

Vitro ubiquitination assay was performed as follows: pACYC-UBA1-UBCH7-UB-HA-HERC3 and pACYC-UBA1-UBCH7-UB-HA-control plasmids were co-transfected with pET28A-His-RPL23A in BL21 *E. coli* cells and further cultured with culture medium supplemented with ampicillin and chloramphenicol antibiotics. Following experiments were available in our previous published work 7.

### Colony formation assay

Respectively, 500 corresponding CRC cells were added each well from a 6-well dish. After 10 days culture, samples were cleaned by PBS, and then fixed under 4% paraformaldehyde (Merck, Kenilworth, USA), and finally stained by 0.1% crystal violet (YEASEN, Shanghai, China). Photographs were taken, and the number of colonies in each cell was then counted.

### CCK-8 assay

CCK-8 (Cell Counting Kit-8 assays) (YEASEN, Shanghai, China) was utilized under the manual. 2000 CRC cells were individually planted into each well of a 96-well plate. The culture condition was as previously described. Serum-free medium supplemented with 10 μL CCK-8 solution was injected to the cells every 24 hours. After 2 hours of incubation, the cells were quantified by spectrophotometry according to the absorbance at 450 nm by a microplate reader (Bio-Rad, Shanghai, China).

### EdU assay

2 × 10^4^ CRC cells were planted into each cell of a 24-well plate. The EdU Apollo®488 In Vitro Imaging Kit (RiboBio, Guangzhou, China) was used to conduct the EdU assays under the manual. Nuclei were stained by DAPI. Photos were taken with a Nikon microscope (Nikon, Japan).

### Analysis of the cell cycle

Cells were cleaned by PBS and then harvested. Then, cells were stained with propidium iodide (KeyGen Biotech, Nanjing, China). Results were detected by flow cytometry.

### In vivo tumor growth assay

BALB/c nu/nu mice (male, aged 6 weeks) were acquired from Shanghai SLAC Laboratory Animal Co. Ltd. In total, 2 × 10^6^ corresponding CRC cells were injected subcutaneously. The Animal Ethics Committee of Zhongshan Hospital Fudan University approved the animal studies. The mice were sacrificed after 3 weeks. The formula: (width^2^ × length)/2 was used to calculate the tumor size.

### Gene Set Enrichment Analysis (GSEA)

GSEA concentrating on RPL23A was based on data from TCGA (COAD and READ). It contained 2 parts: (1) The median expression of RPL23A was set as the cutoff and patients were classified as 2 groups, differential analysis was conducted via “limma” and results were subjected to GSEA; (2) GSEA was performed according to the results obtained from Person correlation analysis concentrating on RPL23A. R language (Version 3.6.2) was used to perform the GSEA. “h: the hallmark gene sets,” that was obtained from MSigDB (https://www.gsea-msigdb.org/gsea/index.jsp), and was set as the gene reference.

### Statistics

Differential gene expression analysis was conducted as mentioned previously. Chi-squared tests were conducted to research the associations between the clinical features and the expression of corresponding proteins. Pearson correlation analysis was performed to explore the expression correlations among diverse proteins. Survival analysis was assessed and visualized via the Kaplan-Meier method and log-rank test, and the results were visualized by means of the R language (Version 3.61). Relevant data was analyzed by Student's t-tests, and the results were shown by GraphPad Prism. * signified P<0.05, ** signified P<0.01, and *** signified P<0.001.

## Results

### HERC3 was identified to be associated with tumor size and could inhibit CRC cell growth and arrest the cell cycle at the G0-G1 phase

**Figure 1:**
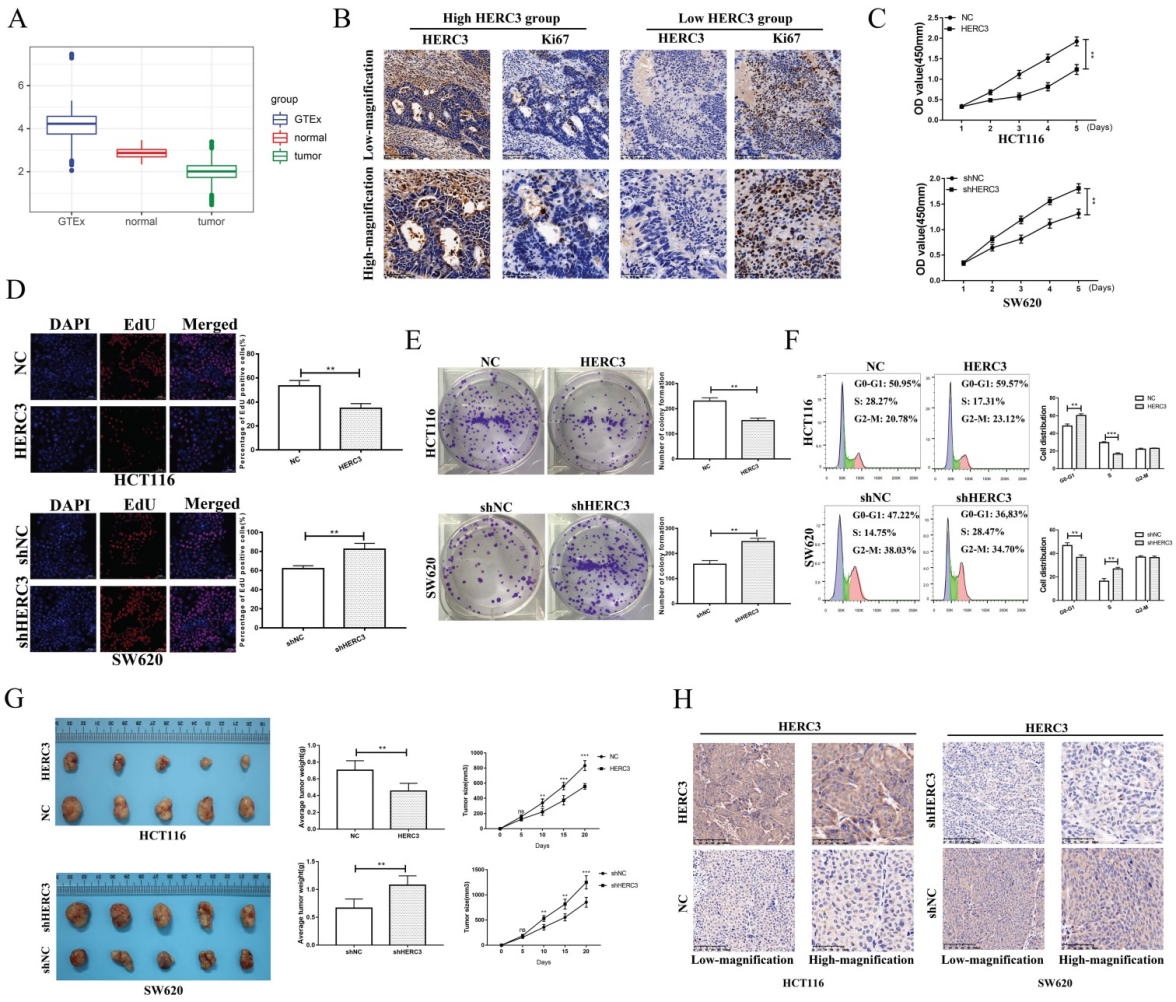
** HERC3 was identified to be associated with tumor size and could inhibit CRC cell growth and blocked the cell cycle at the G0-G1 phase**. (A) Boxplot depicting the gradual decreasing trend from colorectal tissues in healthy individuals to adjacent-tumors normal tissue in CRC patients, and to tumor tissues. (B) Patients with higher HERC3 expression showed lower Ki67 expression. Representative images of HERC3 and Ki67 staining indicating protein expression in the indicated groups (Continuous slices in individual groups and were further subjected to IHC with the indicated antibodies). The median protein expression of HERC3 was set as the cutoff to classify the CRC patients into 2 groups. CCK-8 (C), EdU (D), and colony formation (E) assays proved that HERC3 upregulation inhibited HCT116 cell proliferation, while HERC3 downregulation promoted SW620 cell proliferation, scale bars for views of EdU assays were 50μm. (F) Upregulated HERC3 arrested the cell cycle in the G0-G1 phase in HCT116 cells and induced the opposite effect in SW620 cells. (G) HERC3 upregulation resulted in lighter and smaller tumors and HERC3 downregulation resulted in heavier and bigger tumors in vivo. (H) The expression of HERC3 in tumors formed by HCT116 and SW620 was validated via IHC.

We conducted a 3-times differential analysis as described in section “Materials and methods”. Finally, 11 upregulated and 13 downregulated E3 ligases were screened out (**Supplementary Figure S1A**). The expression heatmap of these 24 differentially expressed E3 ligases was presented in the form of a heatmap (**Supplementary Figure S1B**). According to the survival analysis of these 24 E3 ligases, HERC3 was identified to exert important functions during CRC progression as it exhibited the same trend in the results of differential analysis and survival analysis (HERC3 was gradually decreased in CRC, and HERC3 upregulation indicated better prognostic outcomes). The expression pattern is shown in** Figure 1A**. Interestingly, HERC3 was enriched again and further confirmed our previous published work 7. However, the most featured characteristic for tumorigenesis is uncontrolled cell proliferation. The functions of HERC3 on CRC cell growth are still not clarified. Interestingly, in the present study, HERC3 was firstly found to be associated with tumor size and Ki67 staining, an index for evaluating proliferative capacity of cancer cells (**Table 1**). We additionally found that patients with higher HERC3 expression showed lower Ki67 expression and patients with lower HERC3 expression showed higher Ki67 expression (**Figure 1B**) based on samples from Zhongshan Hospital, Fudan University (another cohort with tumor size information which is totally different from our previous published work) 7. Based on patient samples, it was further confirmed that the protein expression levels of HERC3 in CRC tissue was lower than in tumor-adjacent samples (**Supplementary Figure S2A**). Compared to that in the NCM460 cells, the expression of HERC3 protein was also lower in the CRC cells (HCT116, SW480, DLD-1 and SW620) cells (**Supplementary Figure S2B**). The prognostic value of HERC3 was shown in **Supplementary Figure S2C** according to data in GEPIA2. Two stable cell lines were constructed for further vivo and vitro experiments. For SW620 cells, we knocked down HERC3. For HCT116 cells, we overexpressed HERC3 (**Supplementary Figure S2D**). We used CCK-8, colony formation and EdU assays to research the effects of HERC3 on CRC cell growth. The data proved that HERC3 upregulation could suppress CRC cells proliferation and while HERC3 downregulation could enhance the CRC cells proliferative ability (**Figure 1C, D, E**). Furthermore, HERC3 upregulation arrested the CRC cell cycle in the G0-G1 phase (**Figure 1F**). The effects of HERC3 were further validated in vivo: upregulated HERC3 could result in a lighter and smaller tumor, HERC3 downregulation resulted in heavier and larger tumors (**Figure 1G, the entire view of the vivo experimental mice were shown in Supplementary Figure S2E**). The expression of HERC3 in tumors formed by corresponding stable cell lines (HCT116 and SW620) was validated via IHC (**Figure 1H**).

### Identification of RPL23A as a potential HERC3-interaction protein and exploration of the expression pattern and prognostic value of RPL23A

**Figure 2:**
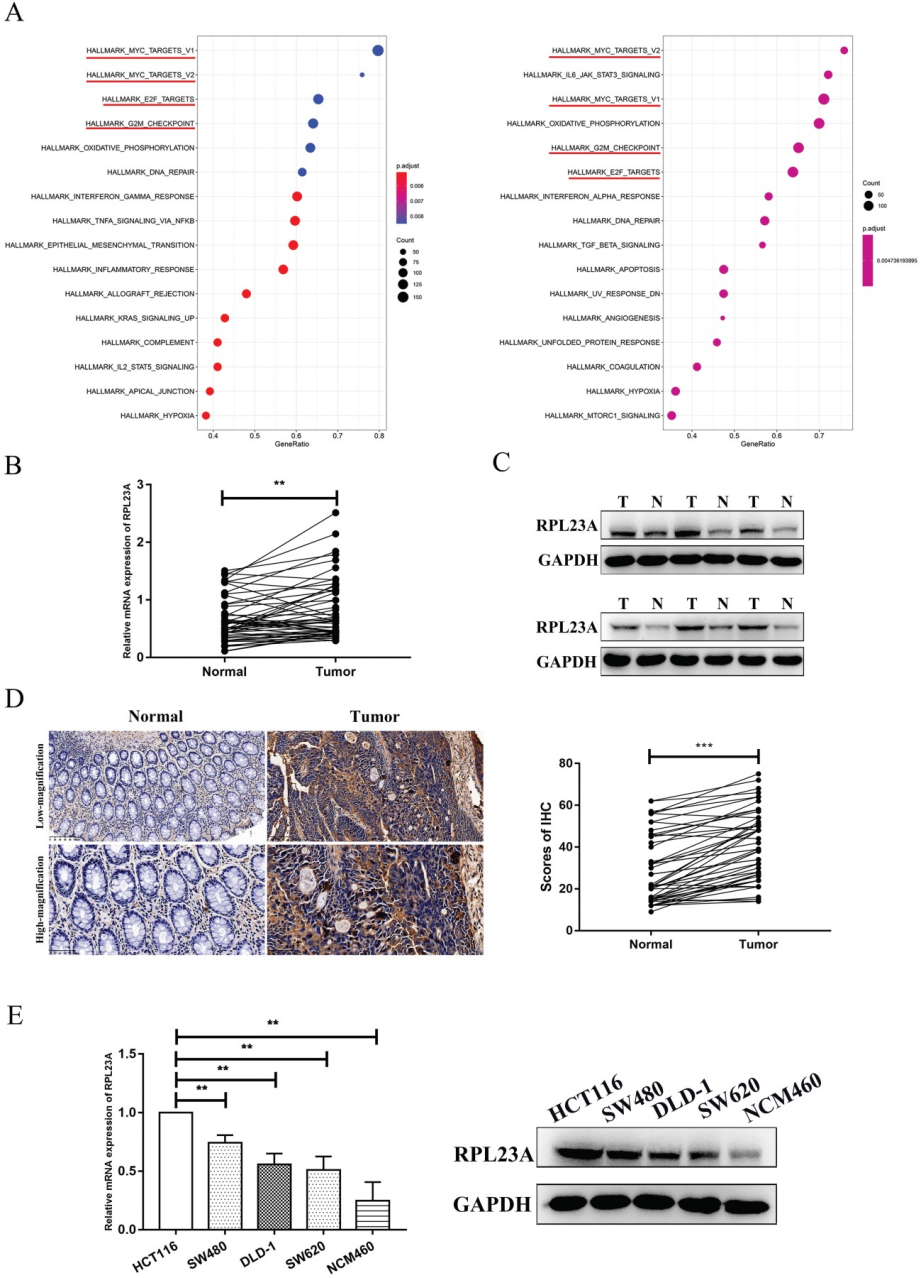
** Identification of RPL23A as a potential HERC3-interaction protein and exploration of the expression pattern and prognostic value of RPL23A**. (A) GSEA results of RPL23A based on data from TCGA (COAD and READ). Left panel: The median protein expression of RPL23A was set as the cutoff, and patients were classified into 2 groups, differential analysis was conducted via “limma” and results were subjected to GSEA. Right panel: GSEA was performed according to the results obtained from Person correlation analysis of RPL23A. (B) Results of qRT-PCR showed that RPL23A mRNA was upregulated in CRC. (C) The protein expression of RPL23A was up-regulated in 6 paired CRCs and CRC-adjacent normal tissues. (D) IHC results based on 50 paired cancer samples and adjacent normal samples indicated that RPL23A was upregulated in CRC, scale bars for low-magnification were 100μm, for high-magnification were 50μm. (E) RPL23A was upregulated in CRC cell lines compared to normal colorectal cells according to the qRT-PCR and western blotting.

We previously identified EIF5A2 as a substrate of HERC3, however, EIF5A2 had almost none functions on CRC cell growth and cell cycle indicating that HERC3 regulated cell growth and cell cycle mainly dependent on other substrate 7. Mass spectrometry analysis and co-IP were performed through HCT116 cells that were transfected with HERC3-Flag plasmids and cells in control groups were transfected with Flag plasmids. According to the experimental results, the representative peptides identified for RPL23A covered 31.43% of all the peptides of RPL23A. The peptides coverage ratio of RPL23A ranked top among all the proteins identified by mass spectrometry analysis. Moreover, mass spectrometry analysis indicated that RPL23A owned a high credibility to interact with HERC3. Besides, results of GSEA indicated that RPL23A might involve in Myc, E2F and G2M checkpoints pathways that were widely reported to have significant functions in tumorigenesis, cell cycle and cell proliferation that might favor to illustrate the functions of HERC3 on CRC cell proliferation and cell cycle (**Figure 2A**). Three representative peptides of RPL23A that were identified through mass spectrometry analysis were presented in the liquid chromatography-tandem mass spectrometry (LC-MS/MS) spectrum (**Supplementary Figure 3**).

In the analysis of 50 tumor-adjacent tissues and paired tumor samples, the RPL23A mRNA expression was upregulated in tumor samples (**Figure 2B**). Protein expression of RPL23A was also validated to be upregulated in CRC samples through western blotting by randomly selecting 6 paired tumor and normal samples (**Figure 2C**) and also further verified according to the results of IHC based on 50 CRC tumor-adjacent normal samples and corresponding matched tumor samples (**Figure 2D**). Moreover, compared to that in the NCM460 cells, the expression of RPL23A mRNA and protein was upregulated in the CRC cells (HCT116, SW480, DLD-1, and SW620) (**Figure 2E**). Based on IHC data and the corresponding clinical information in our center, high RPL23A expression indicated poor OS and DFS outcomes (**Supplementary Figure S4**).

### Validation of RPL23A as a HERC3-interaction protein and identification that the expression of RPL23A is negatively correlated with HERC3

**Figure 3:**
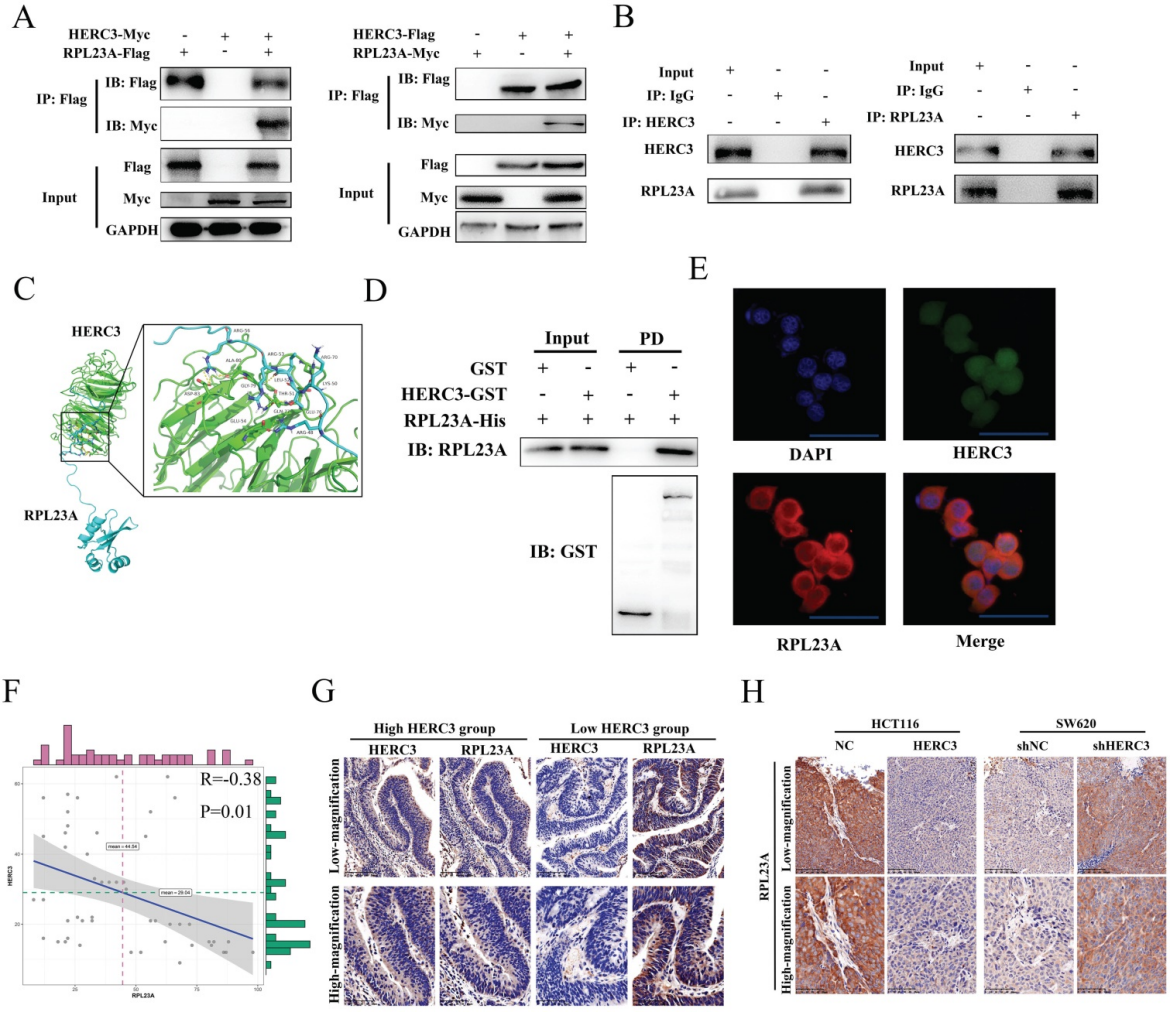
** Verification of RPL23A as a HERC3-interaction protein and identification that the expression of RPL23A is negatively correlated with HERC3.** (A) Exogenously expressed HERC3 and RPL23A could form mutual combination. The indicated plasmids were transfected into HCT116, and samples were then processed to co-IP detection. (B) Endogenous HERC3 can interact with RPL23A, and HCT116 lysates were subjected to IP with anti-HERC3 or anti-RPL23A and control IgG. (C) Detailed amino acid hydrogen combination places between HERC3 and RPL23A were forecasted. Related protein structures were determined by SWISS-MODEL and interaction was performed via ClusPro. (D) RPL23A could interact with HERC3 in vitro. Recombinant HERC3-GST, GST-only, and RPL23A-His were purified from BL21 *Escherichia coli* cells, GST pulldown and western blotting were performed. (E) Exogenous HERC3 and RPL23A could colocalize in HCT116 cells that was detected by immunofluorescence staining, scale bars were 50μm. (F) RPL23A was negatively correlated with HERC3 in 50 CRC tissues according to IHC. (G) Representative images of HERC3 and RPL23A staining indicating protein expression in the indicated groups (continuous slices in individual groups and were further subjected to IHC with the indicated antibodies).The median protein expression of HERC3 was set as the cutoff, and the patients were divided into 2 groups. (H) In vivo assays revealed that tumors in the HERC3-up-regulated group showed relatively low protein levels of RPL23A, while those in the HERC3-down-regulated group showed relatively high protein levels of RPL23A. Scale bars for low-magnification were 100μm, for high-magnification were 50μm.

Further validation experiments were performed. Exogenous RPL23A and HERC3 could interact with each other (**Figure 3A**). Moreover, endogenous HERC3 was indicated to interact with RPL23A and vice versa (**Figure 3B**). The interaction between HERC3 and RPL23A is predicted to depend on hydrogen bonds formed by several specified amino acids (**Figure 3C**). Furthermore, a GST pulldown assay proved that RPL23A could directly bind HERC3 in vitro (**Figure 3D**). HERC3 was further revealed to co-localize with RPL23A in HCT116 cells detected by immunofluorescence assays (**Figure 3E**). Besides, 50 CRC tumor tissues were used to research the correlation between RPL23A and HERC3 through IHC, and the results indicated that there was a negative correlation between HERC3 and RPL23A (**Table 2**, **Figure 3F**). Setting the median expression of HERC3 as the cutoff values, CRC patients were classified into 2 groups, and the patients who owned relatively higher HERC3 protein expression presented relatively low protein levels of RPL23A and vice versa (**Figure 3G**). Moreover, the tumors that formed in vivo experiments validated the expression relationship between HERC3 and RPL23A: tumors formed from the HERC3 upregulation group showed relatively low protein levels of RPL23A compared to that of tumors from control group and vice versa (**Figure 3H**). Besides, taken the median expression of RPL23A and HERC3 together, the 200 patients were classified as 2 groups (patients with high expressed RPL23A+low expressed HERC3 and patients with low expressed RPL23A+high expressed HERC3). Survival analysis was conducted based on these 2 groups of patients, the results indicated that patients with high protein expression level of HERC3 and low protein expression level of RPL23A indicated better OS and DFS clinical outcomes than the patients in the compared group (**Supplementary Figure S5**).

### RPL23A could independently regulated the CRC cell proliferation and cell cycle

**Figure 4:**
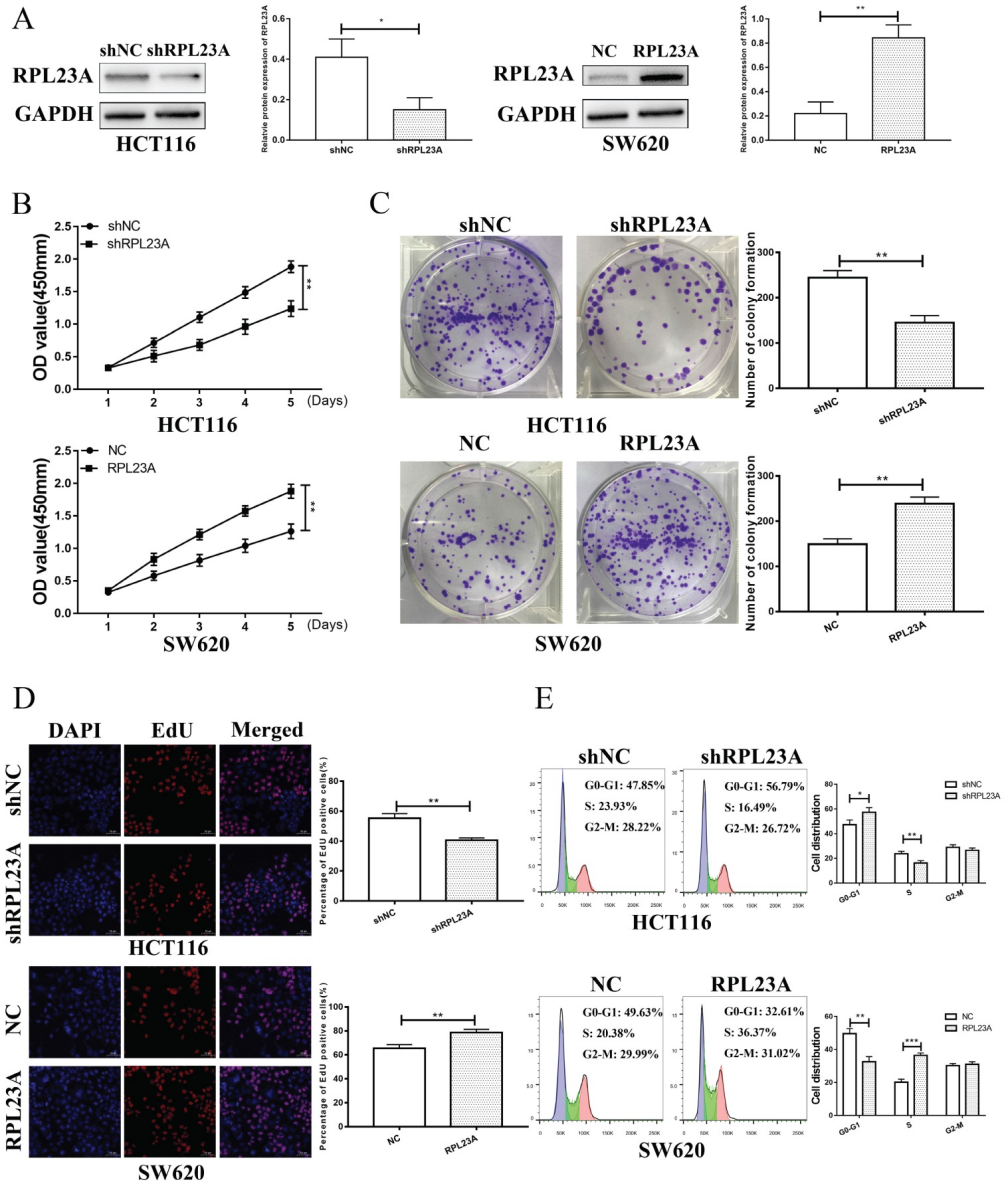
** RPL23A could independently regulated the CRC cell growth and cell cycle.** (A) The efficiency of the indicated lentivirus was validated through western blotting. RPL23A downregulation inhibited HCT116 cell proliferation detected via CCK-8 (B), colony formation (C), and EdU (D) assays, while RPL23A upregulation promoted SW620 cell proliferation. (E) Downregulated RPL23A blocked the cell cycle at G0-G1 phase in HCT116 cells, while upregulation RPL23A induced the opposite effects in SW620 cells. Scale bars for views of EdU assays were 50μm.

To determine whether HERC3 exerts its functions through RPL23A, functions of RPL23A on CRC was firstly researched. The efficiency of the corresponding lentivirus was verified (**Figure 4A**). Through CCK8, EdU, colony formation and cell cycle analysis assays, we discovered that RPL23A downregulation could inhibit CRC cells proliferation and arrest CRC cells in the G0-G1 phase independently and vice versa (**Figure 4B-E**).

### HERC3 exerted biological functions through RPL23A in CRC cells

**Figure 5:**
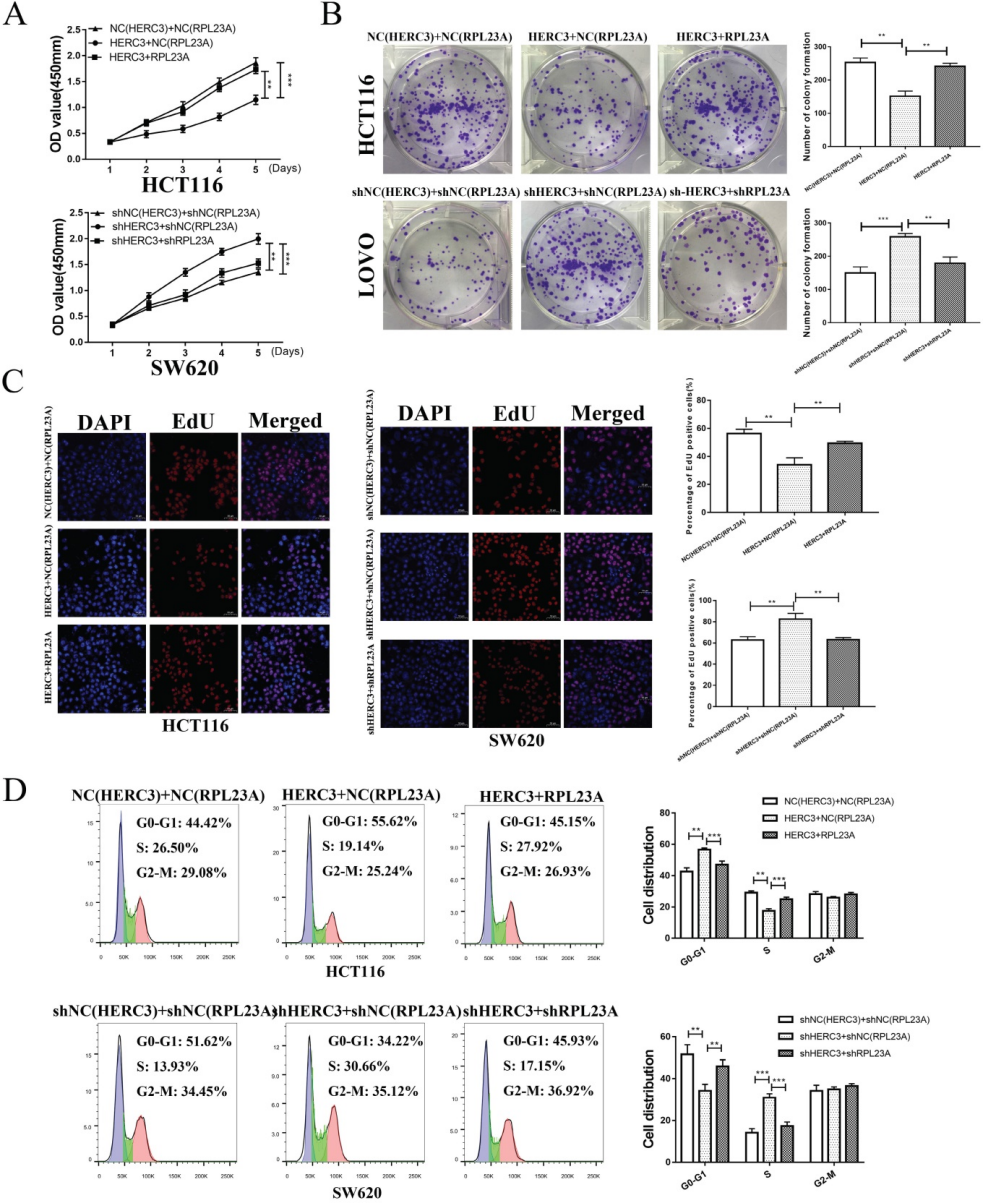
** HERC3 exerted its biological functions via RPL23A in CRC cells.** RPL23A could partially attenuated the functions of HERC3 on CRC cell growth and cell cycle that was proved by CCK-8 (A), colony formation (B), EdU (C) and cell cycle analysis assays (D), HCT116 and SW620 cells were co-infected with corresponding lentivirus. Scale bars for views of EdU assays were 50μm.

The rescue experiments were further carried out. After co-infection, several cell proliferation-related assays including EdU assays, CCK-8, and colony formation were performed, and the results indicated that upregulated RPL23A could attenuate the effects on CRC cell proliferation induced by HERC3 overexpression and vice versa (**Figure 5A, B, C**). In addition, RPL23A attenuated the effects of HERC3 on the cell cycle of CRC (**Figure 5D**). These results indicated that HERC3 exerted biological functions via RPL23A in CRC cell lines.

### HERC3 promoted the proteolytic ubiquitination of RPL23A via the HECT domain at several sites in a K48-dependent linkage

**Figure 6:**
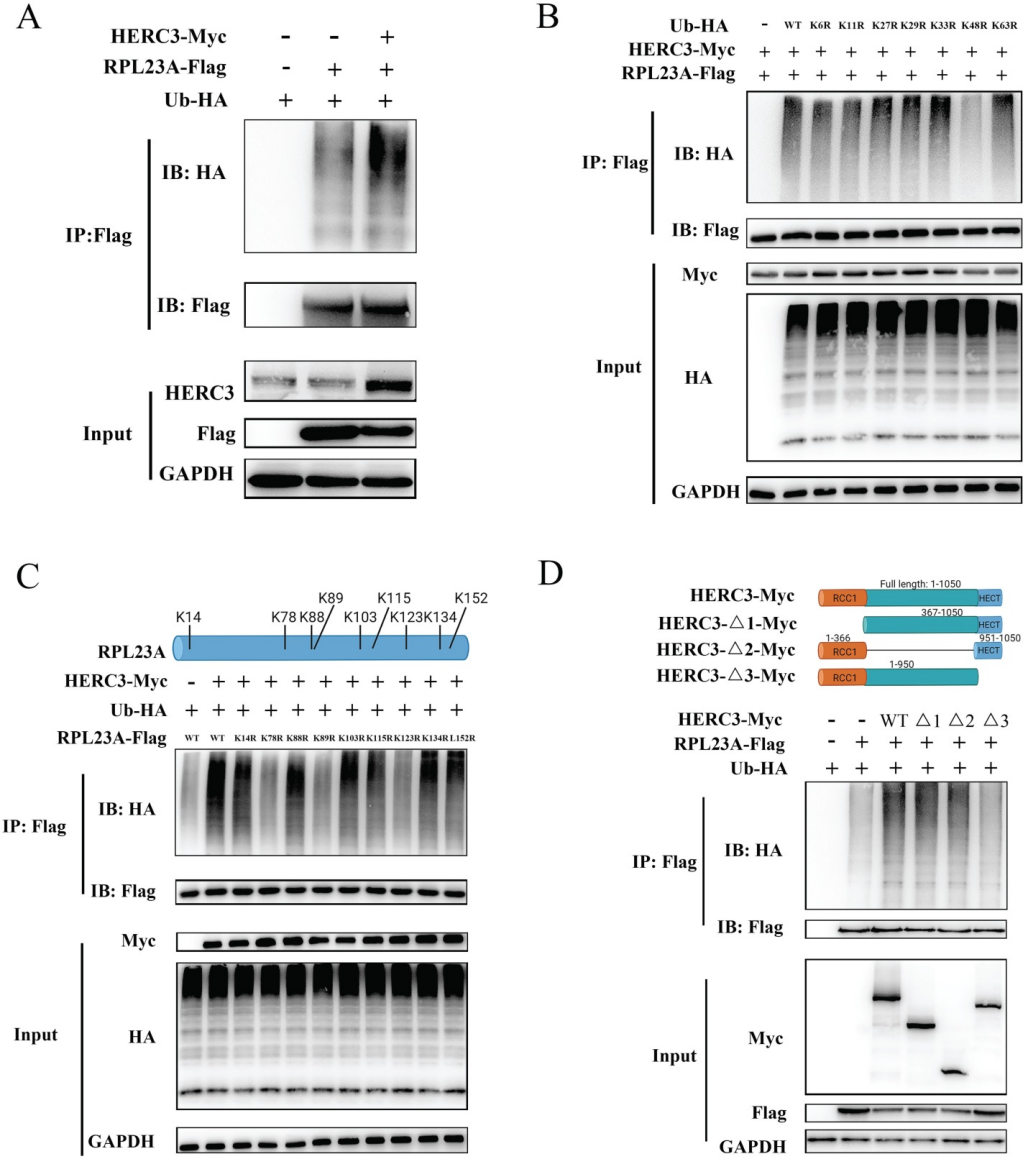
** HERC3 promoted the proteolytic ubiquitination of RPL23A.** (A) HERC3 enhanced the ubiquitination degree of RPL23A in HCT116 cells. In vivo ubiquitylation assays with the indicated plasmids and co-IP were performed. (B) HERC3 combined poly-Ub with RPL23A through the K48 connection. Indicated plasmids were co-transfected into HEK293T and cells were then subjected to co-IP. (C) K78, K89, and K123 on RPL23A were identified as sites where HERC3 transmitted poly-Ub to. HEK293T cells were co-transfected with the indicated plasmids and further subjected to co-IP. (D) HECT structural removal significantly reduced RPL23A ubiquitination. Wild-type HERC3 or several different deletion mutants (shown above) were co-transfected into HEK293T cells with other indicated plasmids.

Given that HERC3 acted as an E3 ligase and HERC3 could interact with RPL23A, we wondered whether RPL23A was ubiquitinated by HERC3. Results of in vivo ubiquitylation assay showed that HERC3 overexpression resulted in significant RPL23A polyubiquitination in HCT116 cells (**Figure 6A**). Seven HA-Ub mutants were co-transfected into HEK293T with the indicated plasmids. The results of vivo ubiquitylation assays demonstrated that poly-Ub might be conjugated to RPL23A via K48 linkage, which might target substrates for proteasome-dependent degradation (**Figure 6B**). Moreover, in HCT116, we revealed that transfection of HERC3-Flag plasmid could induce the degradation of RPL23A via a dose-dependent manner (**Supplementary Figure S6A**). The protein levels of RPL23A increased following treatment with MG-132, indicating that RPL23A is regulated via a ubiquitin-proteasome pathway. In addition, we discovered that MG-132 could restore the degradation of RPL23A, which was mediated by HERC3 in HCT116 cells (**Supplementary Figure S6B**). Furthermore, CHX chase assays proved that HERC3 overexpression could decrease the half-life of RPL23A in HCT116 cells (**Supplementary Figure S6C**). These results suggested that HERC3 might promote the ubiquitination and degradation of RPL23A through a proteasome-dependent pathway.

To investigate the detailed ubiquitination-mediated regulatory mechanism between HERC3 and RPL23A, further studies were conducted. Mass spectrometry analysis showed that 11 Lys residues of RPL23A could be identified as possible side chains where HERC3 transmitted poly-Ub to (**Supplementary Figure S7A-K, and Supplementary Table S1**). Nine Lys residues with top scores were selected for further validation because these residues owned higher credible scores and were more reliable. Therefore, nine individual RPL23A mutants with K-to-R changes were constructed and subjected to in vivo ubiquitination experiments. The results indicated that HERC3-mediated ubiquitylation took place on K78, K89, and K123 of RPL23A (**Figure 6C**). As shown in **Figure 6D**, corresponding plasmids with wild-type HERC3 or several different deletion mutants were co-transfected into HEK293T. In vivo ubiquitylation assays were performed, and the results demonstrated that removal of the HECT structural remarkably reduced the ubiquitination degree of RPL23A.

### HERC3 regulated the protein expression of c-Myc and p21 via RPL23A and could control ubiquitination of p21 via RPL23A

**Figure 7:**
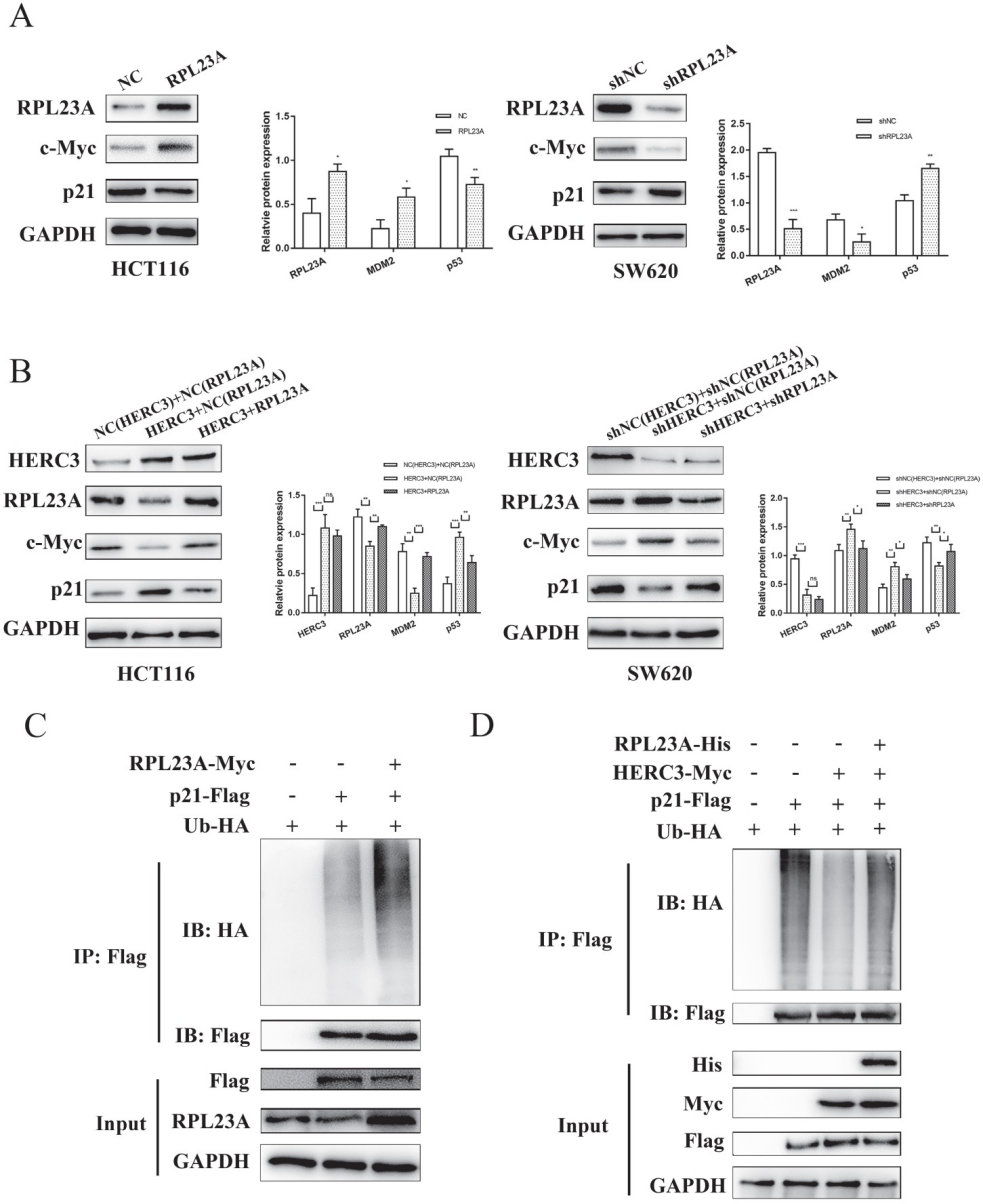
** HERC3 regulated the protein expression of c-Myc and p21 via RPL23A and could control ubiquitination of p21 via RPL23A.** (A) RPL23A regulated the protein expression of c-Myc and p21 in HCT116 and SW620. (B) HERC3 regulated the protein expression of c-Myc and p21, and the effects were partially reversed by RPL23A in HCT116 and SW620. The indicated lentivirus was co-infected HCT116 and SW620, and cell lysates were subjected to western blot. (C) RPL23A was found to enhance the ubiquitination degree of p21 in HCT116 cells. (D) HERC3 reduced the ubiquitination of p21 in HCT116 cells, and RPL23A partially rescued the effects. In vivo ubiquitination assays were performed in HCT116 cells using the plasmids.

According to the results of GSEA (**Figure 2A**), RPL23A was identified to involve in regulating Myc, E2F targets, and G2M checkpoints pathways. We attempted to research the regulatory mechanism of RPL23A via detecting several markers involved in the pathways mentioned above. Interestingly, we discovered that RPL23A could modulate the protein expression of c-Myc and p21 (**Figure 7A**), HERC3 was also verified to modulate the protein expression of c-Myc and p21, and the effects could be partially reversed by RPL23A (**Figure 7B**).

Moreover, in the present study, we found that RPL23A could regulate the ubiquitination of p21 (**Figure 7C**). Interestingly, HERC3 could also influence the ubiquitination of p21 in HCT116 cells, and RPL23A could partially attenuate the effects regulated by HERC3 (**Figure 7D**).

## Discussion

Colorectal cancer (CRC) ranks top among various cancer types in terms of mortality and morbidity 1. The treatment and diagnosis of CRC progress significantly in recent years. However, specific and valuable biomarker for precise treatment and diagnosis remains rare; moreover, the potential mechanism underlying CRC remains unclarified. Thus, it is urgent and essential to find valuable biomarkers to elucidate the underlying mechanism to improve the treatment and diagnosis of CRC.

The UPS exerts fundamental functions, including regulating cellular homeostasis, cell cycle, and cellular metabolism, and even governs cell fate decisions, including cell death, senescence, differentiation, and proliferation. Briefly, the most well-known function of the UPS is that it facilitates the ubiquitination degradation of functional proteins which were identified as substrates. However, perturbations of the UPS can also affect homeostasis, leading to the occurrence of diseases, such as cancer 13; 14. Ubiquitination is characterized by cascade reactions collectively induced by ubiquitin-activating enzymes ubiquitin-conjugating enzymes, and ubiquitin ligases (E3s). E3 ligases are wildly identified as the key members in this cascade that recruit ubiquitin-loaded E2s, recognize specific substrates, and finally catalyze ubiquitin to transfer to the indicated substrate lysine residues. Growing evidence has proven that E3 ligases are important regulators in the development of cancers and can influence the cancer therapeutic response. Thus, E3 ligases can work as promising targets for the exploration of anticancer drugs and can be valuable biomarkers to help to diagnose or elucidate the mechanism of cancers.

In the present study, we performed 3-steps differential analysis based on online database in order to identify the E3s which has gradient expression trend from the colorectal tissues of normal people to the CRC tumor-adjacent tissues, and then to tumor tissues. These E3s could play crucial roles during the entire process of tumorigenesis of CRC and HERC3 was screened out finally. Consistent to our previous work, HERC3 was found to be downregulated in CRC and HERC3 downregulation predicted worse clinical outcomes including OS and DFS. In our previous work, HERC3 was once identified to influence the metastasis of CRC 7. However, one of most featured characteristics for tumorigenesis is the uncontrolled cell growth, and the functions of HERC3 on CRC cell proliferation are still blank. Interestingly, we here discovered that HERC3 protein expression was associated with tumor size and Ki67 staining, an index for evaluating proliferative capacity of cancer cells, indicating that HERC3 might also affect CRC cell proliferation.

HERC3 is a member of HERC E3 family, and there are totally six members in this E3 subgroup. The E3 ligases in this subgroup can be further divided into 2 groups: two huge HERC E3s and four mini HERC E3s. Some members are recognized to exert crucial roles in various cancers. HERC1 was reported to mediate the metastasis of breast cancer cells 8. HERC2 was reported to control the p53/MDM2 axis 9. HERC4 could induce breast cancer progression by downregulating the tumor suppressor LATS1 10. HERC5 was reported to be a latent prognostic index in breast cancer 11. However, researches demonstrating the effects of HERC3 on cancers are still rare, and it was demonstrated that HERC3 could mediate the ubiquitination degradation of SMAD7, further enhance autophagy-mediated epithelial-mesenchymal transition (EMT) and facilitate chemoresistance in glioblastoma 15. In CRC, HERC3 used to be illustrated to have none mutations in CRC and also was once reported to inhibit the metastasis of CRC by targeting EIF5A2 and further controlled the epithelial-mesenchymal transition 7; 16. However, the effects of HERC3 on CRC cell proliferation are unclarified.

Here, we revealed that HERC3 suppressed proliferation of CRC cells in vitro and in vivo, and we demonstrated that HERC3 blocked the cell cycle in G0-G1 phase in CRC. RPL23A was for the first time identified as the direct substrate of HERC3. Moreover, RPL23A was also revealed for the first time to be upregulated in CRC, besides, RPL23A upregulation predicted worse prognostic results, and RPL23A could independently regulate the CRC cell proliferation and cell cycle, furthermore, RPL23A could also partially attenuate the effects of HERC3 indicating that HERC3 might function through RPL23A. Mechanistically, RPL23A could be ubiquitination degraded by HERC3 based on the K48 linkage, and K48 is also commonly identified as the typical type of poly-Ub chain that result in the substrate proteasome degradation 17-19. K78, K89, and K123 on RPL23A were mainly responsible for HERC3-mediated ubiquitylation. Our data suggested that the HECT domain in HERC3 played the crucial role in regulating RPL23A ubiquitination. The detailed regulatory mechanism was demonstrated as HERC3 mediates the proteolytic ubiquitination of RPL23A via the HECT domain at multiple sites (K78, K89, and K123) of RPL23A.

RPL23A was indicated to be involved in Myc, E2F targets and G2M checkpoints pathways according to the results of GSEA. Notably, through detecting several markers of the signaling pathways above, we found that RPL23A could regulated the protein expression of c-Myc and p21. Moreover, HERC3 was revealed to regulate c-Myc/p21 via RPL23A, and HERC3 could also regulate the ubiquitination of p21 via RPL23A.

Myc signaling pathway is widely recognized as one member of the most commonly activated oncogenic pathways in diverse cancers and Myc is also one target for therapy 20. C-Myc is a member of MYC family. In normal conditions, it is controlled by growth factor-dependent pathways, however, c-Myc can also function as a oncogene which is upregulated in diverse kinds of cancers^21^; ^22^. The dysregulation of c-Myc can result from many biological processes such as gene amplification, loss of upstream repressors which function to maintain the expression of c-Myc^22^. In different kinds of cancers including CRC, c-Myc can function as crucial regulator of malignant transformation and regulate many processes including cell proliferation, cell growth, and genomic instability. Moreover, c-Myc is also identified as a therapeutic target for tumor treatment^23^. Hence, the HERC3/RPL23A regulatory axis may also aid the therapy targeting c-Myc. Besides, interferon gamma response and interferon alpha were enriched in the results of GSEA concentrating RPL23A indicating that RPL23A might have significant roles in immune-related pathways, besides, therapies target Myc could also enhance the immunotherapy in cancers 24. These findings also indicated that HERC3/RPL23A axis exerted critical functions in the field of immunity of cancers that provided us a novel research direction. Given the gradually decreasing expression trend in HERC3 expression from healthy colorectal samples to CRC patients' tumor-adjacent normal samples, and then to CRC samples, HERC3 might be involved throughout the initiation and progression of CRC, uncontrolled cell proliferation is characterized as one most common feature of cancer initiation and progression. Furthermore, cell proliferation mainly depends on the control of cell cycle. As a cyclin-dependent kinase inhibitor, p21 is widely recognized as a cell cycle regulator and also revealed to serve a fighter against dysregulated cells that may become cancerous 25. p21 is a cyclin-dependent kinase inhibitor, it can interact with cyclin-cyclin-dependent kinase2 or cyclin-dependent kinase4 complexes and further regulate the cell cycle at G1 phase. Moreover, p21 is tightly controlled by p53, a famous tumor suppressor, thus p21 can regulate the cell cycle especially at the G1 phase in a p53-dependent manner. In the present research, HERC3 was found to reduce the ubiquitination of p21 via RPL23A, moreover, HERC3 could enhance the protein expression of p21. Thus, HERC3 could serve as a rigorous tumor suppressor through RPL23A/c-Myc/p21 axis. These findings might help to invent target antitumor drugs.

In conclusion, HERC3 controlled CRC proliferation, the cell cycle and regulated the c-Myc/p21 axis via directly targeting RPL23A for ubiquitination degradation. This study may provide a new perspective on the treatment and diagnosis of colorectal cancer.

## Supplementary Material

Supplementary figures and table.

## Figures and Tables

**Table 1:** Expression of HERC3 was correlated with tumor size and ki67 staining in 200 CRC patients. Patients were classified into 2 groups and the median protein expression of HERC3 was set as the cutoff.

Characteristics	Number	HERC3 expression		P-value
		High	Low	
Age				0.67
≥60	118	61	57	
<60	82	39	43	
Gender				1
Female	81	41	40	
Male	119	59	60	
Tumor size				
≥4	117	43	74	<0.001
<4	83	57	26	
Ki67				0.02
>50%	158	72	86	
≤50%	42	28	14	

**Table 2:** Results of the chi-squared test proved that HERC3 correlated with RPL23A.

	Total	HERC3 expression		P-value
		High	Low	
RPL23A expression				0.02
High	25	8	17	
Low	25	17	8	

## Abbreviations

CRC: Colorectal cancer
OS: Overall survival
IHC: Immunohistochemistry
IP: Immunoprecipitation
DFS: Disease-free survival
IF: Immunofluorescence
TCGA: The Cancer Genome Atlas
UPS: Ubiquitin proteasome system
E3s: Ubiquitin ligases
GSEA: Gene Set Enrichment Analysis
GTEx: Genotype-Tissue Expression
Co-IP: Coimmunoprecipitation
CHX: Cycloheximide
FDR: False discovery rate
qRT-PCR: Quantitative reverse transcription-polymerase chain reaction

## References

[1] Siegel R L, Miller K D, Goding Sauer A, Fedewa S A, Butterly L F, Anderson J C (2020). Colorectal cancer statistics, 2020. CA: a cancer journal for clinicians.

[2] Zheng N, Shabek N (2017). Ubiquitin Ligases: Structure, Function, and Regulation. Annu Rev Biochem.

[3] Pickart C M (2001). Mechanisms underlying ubiquitination. Annu Rev Biochem.

[4] Buetow L, Huang D T (2016). Structural insights into the catalysis and regulation of E3 ubiquitin ligases. Nature reviews Molecular cell biology.

[5] Lipkowitz S, Weissman A M (2011). RINGs of good and evil: RING finger ubiquitin ligases at the crossroads of tumour suppression and oncogenesis. Nature reviews Cancer.

[6] Rotin D, Kumar S (2009). Physiological functions of the HECT family of ubiquitin ligases. Nature reviews Molecular cell biology.

[7] Zhang Z, He G, Lv Y, Liu Y, Niu Z, Feng Q (2022). HERC3 regulates epithelial-mesenchymal transition by directly ubiquitination degradation EIF5A2 and inhibits metastasis of colorectal cancer. Cell death & disease.

[8] Rossi F A, Calvo Roitberg E H, Enriqué Steinberg J H, Joshi M U, Espinosa J M, Rossi M (2021). HERC1 Regulates Breast Cancer Cells Migration and Invasion. Cancers (Basel).

[9] García-Cano J, Sánchez-Tena S, Sala-Gaston J, Figueras A, Viñals F, Bartrons R (2020). Regulation of the MDM2-p53 pathway by the ubiquitin ligase HERC2. Mol Oncol.

[10] Xu Y, Ji K, Wu M, Hao B, Yao K T, Xu Y (2019). A miRNA-HERC4 pathway promotes breast tumorigenesis by inactivating tumor suppressor LATS1. Protein & cell.

[11] Tang J, Yang Q, Cui Q, Zhang D, Kong D, Liao X (2020). Weighted gene correlation network analysis identifies RSAD2, HERC5, and CCL8 as prognostic candidates for breast cancer. J Cell Physiol.

[12] Ge Z, Leighton J S, Wang Y, Peng X, Chen Z, Chen H (2018). Integrated Genomic Analysis of the Ubiquitin Pathway across Cancer Types. Cell reports.

[13] Mani A, Gelmann E P (2005). The ubiquitin-proteasome pathway and its role in cancer. J Clin Oncol.

[14] Qi J, Ronai Z A (2015). Dysregulation of ubiquitin ligases in cancer. Drug resistance updates: reviews and commentaries in antimicrobial and anticancer chemotherapy.

[15] Li H, Li J, Chen L, Qi S, Yu S, Weng Z (2019). HERC3-Mediated SMAD7 Ubiquitination Degradation Promotes Autophagy-Induced EMT and Chemoresistance in Glioblastoma. Clin Cancer Res.

[16] Yoo N J, Park S W, Lee S H (2011). Frameshift mutations of ubiquitination-related genes HERC2, HERC3, TRIP12, UBE2Q1 and UBE4B in gastric and colorectal carcinomas with microsatellite instability. Pathology.

[17] Iwai K, Tanaka K (2014). Ubiquitin chain elongation: an intriguing strategy. Molecular cell.

[18] Hershko A, Ciechanover A (1998). The ubiquitin system. Annu Rev Biochem.

[19] Kerscher O, Felberbaum R, Hochstrasser M (2006). Modification of proteins by ubiquitin and ubiquitin-like proteins. Annual review of cell and developmental biology.

[20] Bild A H, Yao G, Chang J T, Wang Q, Potti A, Chasse D (2006). Oncogenic pathway signatures in human cancers as a guide to targeted therapies. Nature.

[21] Dong Y, Tu R, Liu H, Qing G (2020). Regulation of cancer cell metabolism: oncogenic MYC in the driver's seat. Signal transduction and targeted therapy.

[22] Gabay M, Li Y, Felsher D W (2014). MYC activation is a hallmark of cancer initiation and maintenance. Cold Spring Harbor perspectives in medicine.

[23] Llombart V, Mansour M R (2022). Therapeutic targeting of "undruggable" MYC. EBioMedicine.

[24] Han H, Jain A D, Truica M I, Izquierdo-Ferrer J, Anker J F, Lysy B (2019). Small-Molecule MYC Inhibitors Suppress Tumor Growth and Enhance Immunotherapy. Cancer Cell.

[25] Sturmlechner I, Zhang C, Sine C C, van Deursen E J, Jeganathan K B, Hamada N (2021). p21 produces a bioactive secretome that places stressed cells under immunosurveillance. Science (New York, NY).

