# Supplementary material for: HERC3 directly targets RPL23A for ubiquitination degradation and further regulates Colorectal Cancer proliferation and the cell cycle
Source: Int J Biol Sci. 2022 May 1;18(8):3282–97. doi: 10.7150/ijbs.72014 (PMC9134906; doi:10.7150/ijbs.72014)
Supplement: Supplementary file 1 — Supplementary figures and table. [file ijbsv18p3282s1.pdf]

Figure legends:

Supplementary Figure S1. Identification of HERC3 as an important role during CRC progression. (A) Venn plots depicting the intersection of aberrantly expressed genes in the upregulated and downregulated groups. (B) Heatmap demonstrating the expression patterns of 24 differently expressed E3 ligase genes in different populations.

Supplementary Figure S2. Expression pattern and prognostic value of HERC3. (A) IHC results based on 50 paired CRCs and CRC-adjacent normal samples indicated that HERC3 protein expression was downregulated in CRC samples, scale bars for low-magnification were 100μm, for high-magnification were 50μm. (B) HERC3 protein expression was lower in CRC cells than normal cells according to western blotting. (C) HERC3 was found to be associated with prognostic outcomes, including OS and DFS conducted through GEPIA2. The median protein expression of HERC3 was set as the cutoff and the CRC patients were classified into 2 groups. (D) Construction of stable HCT116 or SW620 CRC cell lines with upregulated HERC3 or downregulated HERC3 expression by infection with the indicated lentivirus. (E) The entire view of the vivo experimental mice.

Supplementary Figure S3. Representative peptides identified by mass spectrometry analysis were presented in spectra of liquid chromatography-tandem mass spectrometry (LC-MS/MS).

Supplementary Figure S4. Survival analysis of RPL23A. Upregulation RPL23A indicated poor OS and DFS based on data from our center. The median protein expression of RPL23A was set as the cutoff and patients were classified into 2 groups.

Supplementary Figure S5. Survival analysis of patients who were classified according to the median protein expression of HERC3 and RPL23A. Patients with high protein expression level of HERC3 and low protein expression level of RPL23A showed better

OS and DFS results than patients with low protein expression level of HERC3 and high protein expression level of RPL23A.

Supplementary Figure S6. HERC3 degraded RPL23A in a ubiquitin proteasome dependent manner. (A) In HCT116, ectopic expression of HERC3-Flag induced the degradation of RPL23A in a dose-dependent manner. (B) RPL23A was validated to be degraded in a ubiquitin-proteasome-dependent manner under MG-132 treatment when HCT116 were transfected with the corresponding plasmids. (C) CHX chase assays proved that HERC3 overexpression could decrease the RPL23A half-life in HCT116.

Supplementary Figure S7. Peptides identified by mass spectrometry analysis for the identification of the ubiquitination modification sites were presented in spectra of liquid chromatography-tandem mass spectrometry (LC-MS/MS). Figures are ordered by (A)-(K) according to the relevant score in Supplementary Table S1.

Supplementary Table S1. Peptides identified by mass spectrometry analysis for the identification of ubiquitination modification sites.

Sequence	Score
IEDNNTLVFIVDVK	124.74
KIEDNNTLVFIVDVK	109.38
LAPDYDALDVANK	76.31
VNTLIRPDGEK	70.08
KLYDIDVAK	63.98
FPLTTESAMK	58.44
KEAPAPPK	53.57
KEAPAPPKAEAK	47.2
NKLDHYAIIK	38.91
LYDIDVAK	38.27
LDHYAIIK	31.45

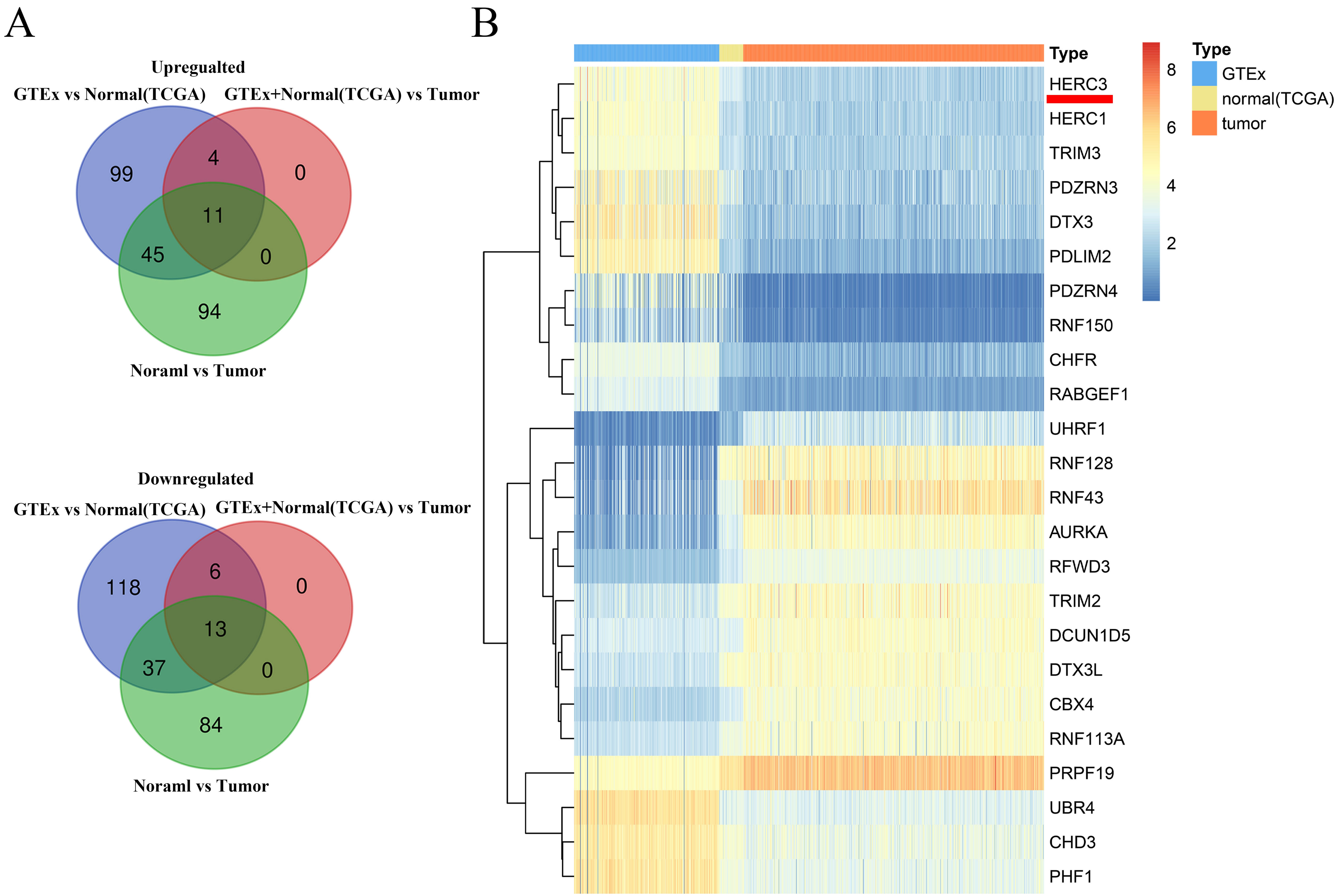
A
B
Upregualted
GTEx vs Normal(TCGA)
GTEx+Normal(TCGA) vs Tumor
99
4
0
11
45
0
94
Noraml vs Tumor
Downregulated
GTEx vs Normal(TCGA)
GTEx+Normal(TCGA) vs Tumor
118
6
0
13
37
0
84
Noraml vs Tumor
Type
HERC3
HERC1
TRIM3
PDZRN3
DTX3
PDLIM2
PDZRN4
RNF150
CHFR
RABGEF1
UHRF1
RNF128
RNF43
AURKA
RFWD3
TRIM2
DCUN1D5
DTX3L
CBX4
RNF113A
PRPF19
UBR4
CHD3
PHF1
8
6
4
2
Type
GTEx
normal(TCGA)
tumor

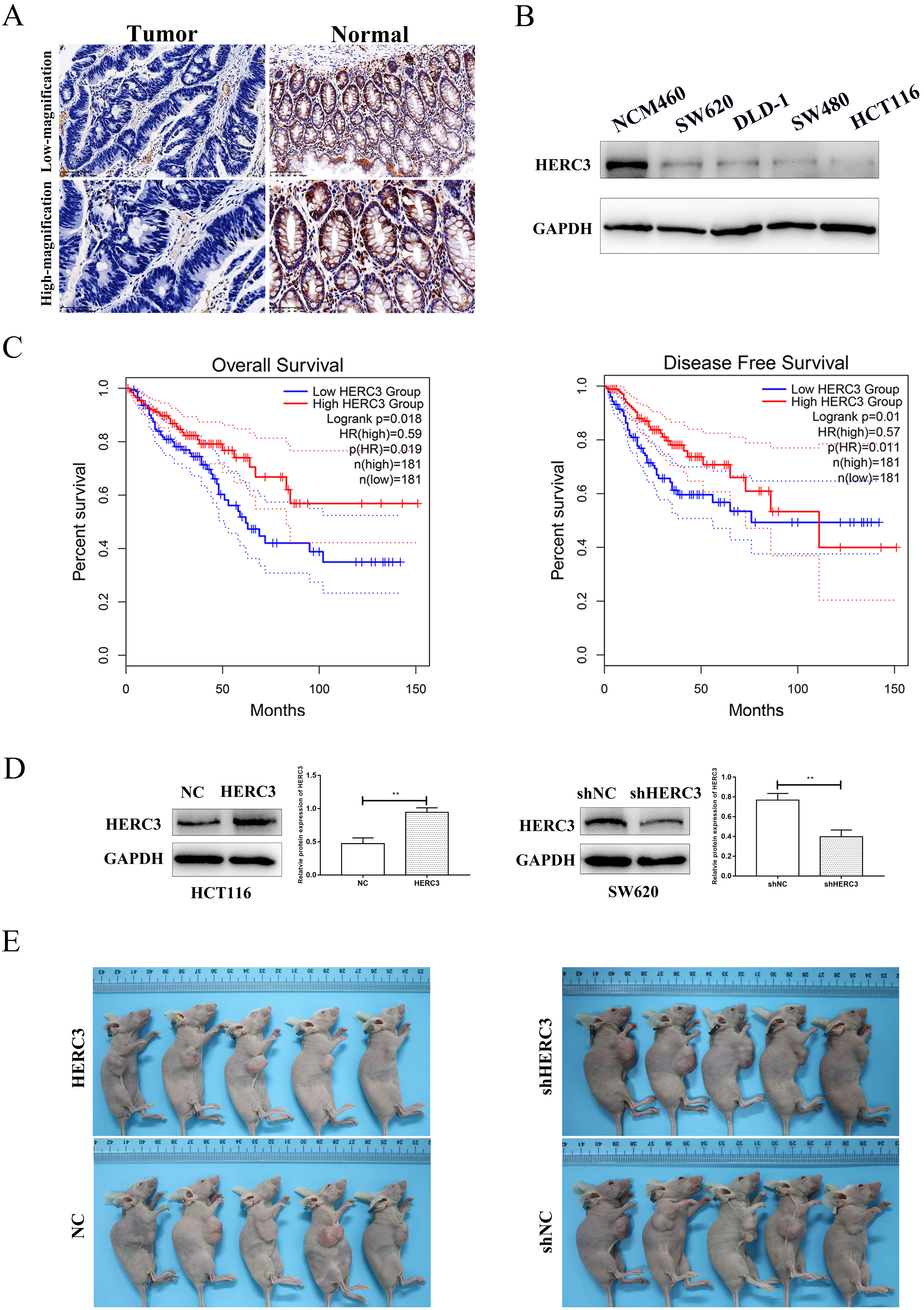
A
Tumor
Normal
Low-magnification
High-magnification
B
NCM460
SW620
DLD-1
SW480
HCT116
HERC3
GAPDH
C
Overall Survival
Low HERC3 Group
High HERC3 Group
Logrank p=0.018
HR(high)=0.59
p(HR)=0.019
n(high)=181
n(low)=181
Percent survival
Months
Disease Free Survival
Low HERC3 Group
High HERC3 Group
Logrank p=0.01
HR(high)=0.57
p(HR)=0.011
n(high)=181
n(low)=181
Percent survival
Months
D
NC
HERC3
HERC3
GAPDH
HCT116
Relatvie protein expression of HERC3
**
NC
HERC3
shNC
shHERC3
HERC3
GAPDH
SW620
Relatvie protein expression of HERC3
**
shNC
shHERC3
E
HERC3
NC
shHERC3
shNC

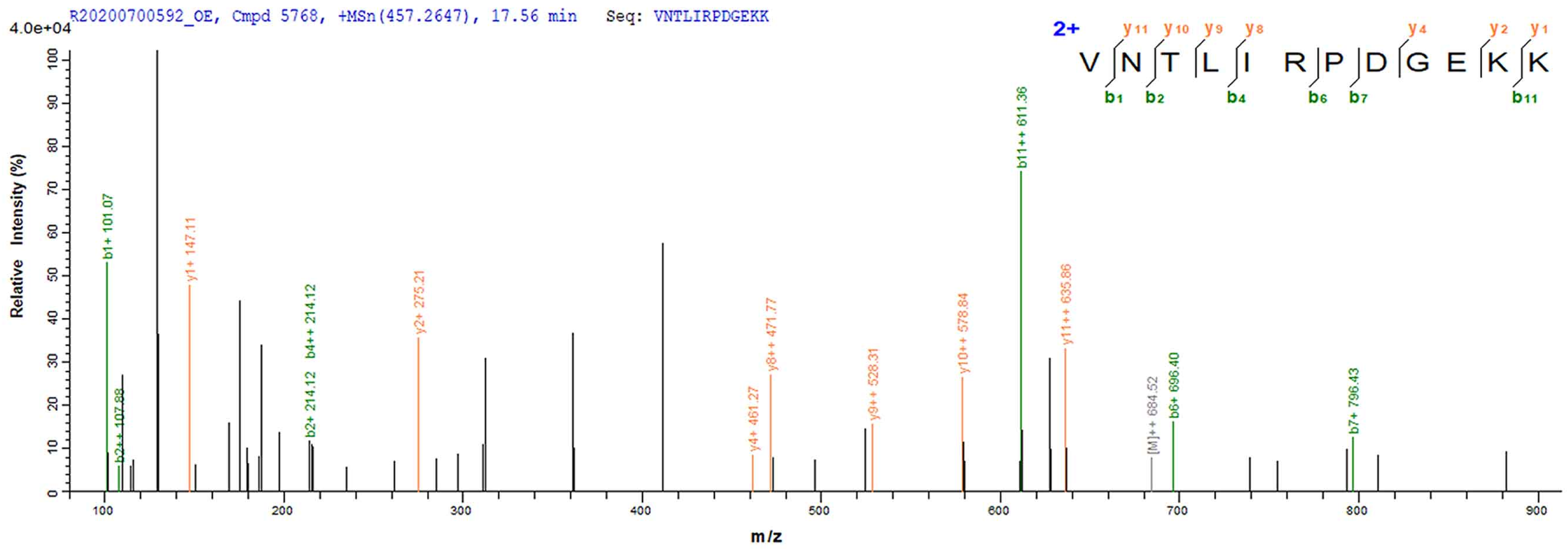
R20200700592_OE, Cmpd 5768, +MSn(457.2647), 17.56 min Seq: VNTLIRPDGEKK
4.0e+04
2+
VNTLIRPDGEKK
Relative Intensity (%)
m/z

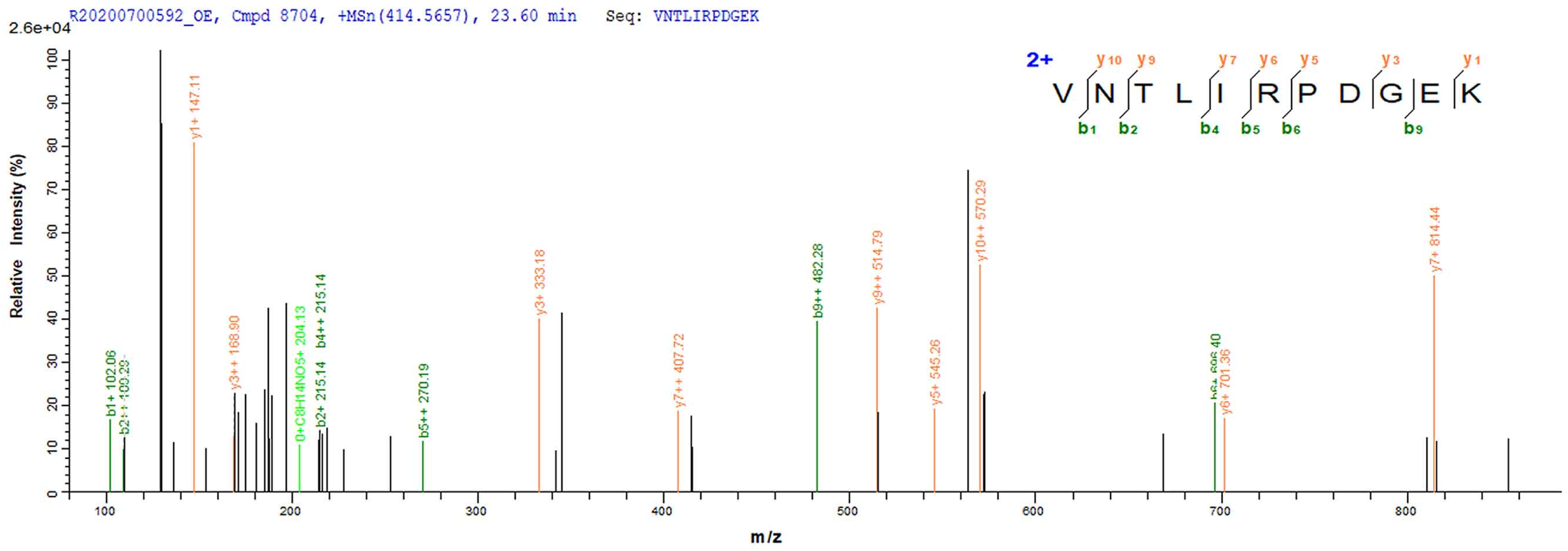
R20200700592_OE, Cmpd 8704, +MSn(414.5657), 23.60 min Seq: VNTLIRPDGEK
2.6e+04
2+
VNTLIRPDGEK
Relative Intensity (%)
m/z

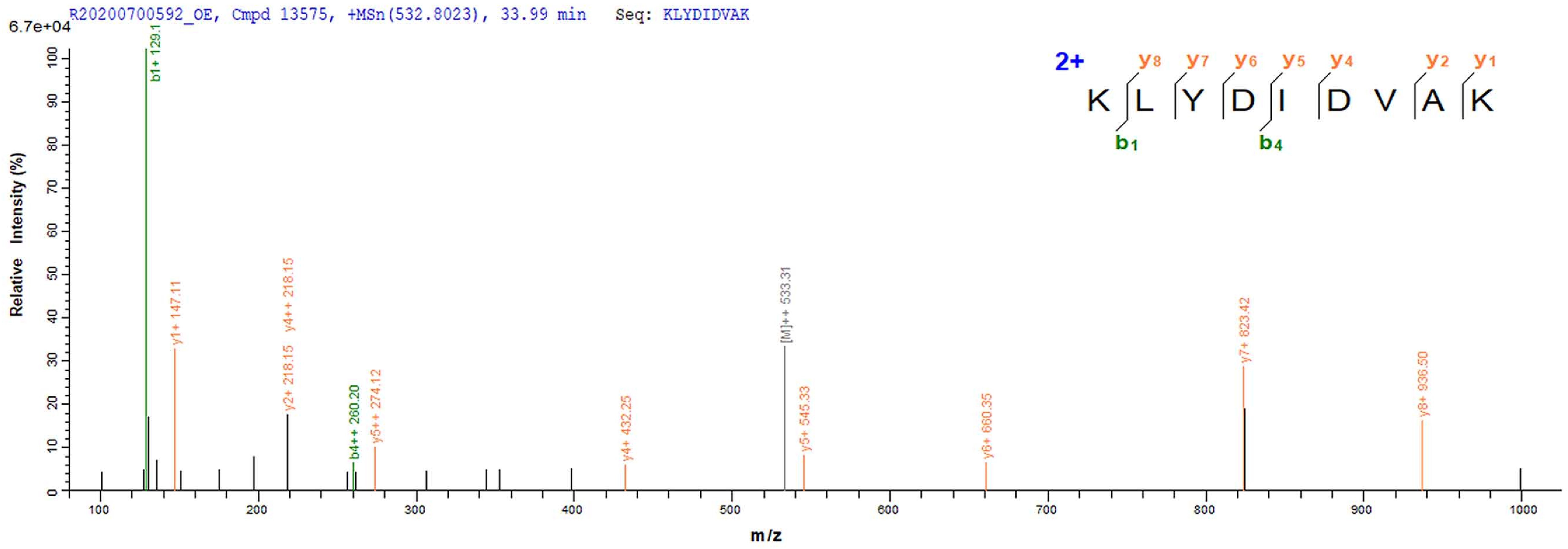
R20200700592_OE, Cmpd 13575, +MSn(532.8023), 33.99 min Seq: KLYDIDVAK
6.7e+04
2+
KLYDIDVAK
Relative Intensity (%)
m/z

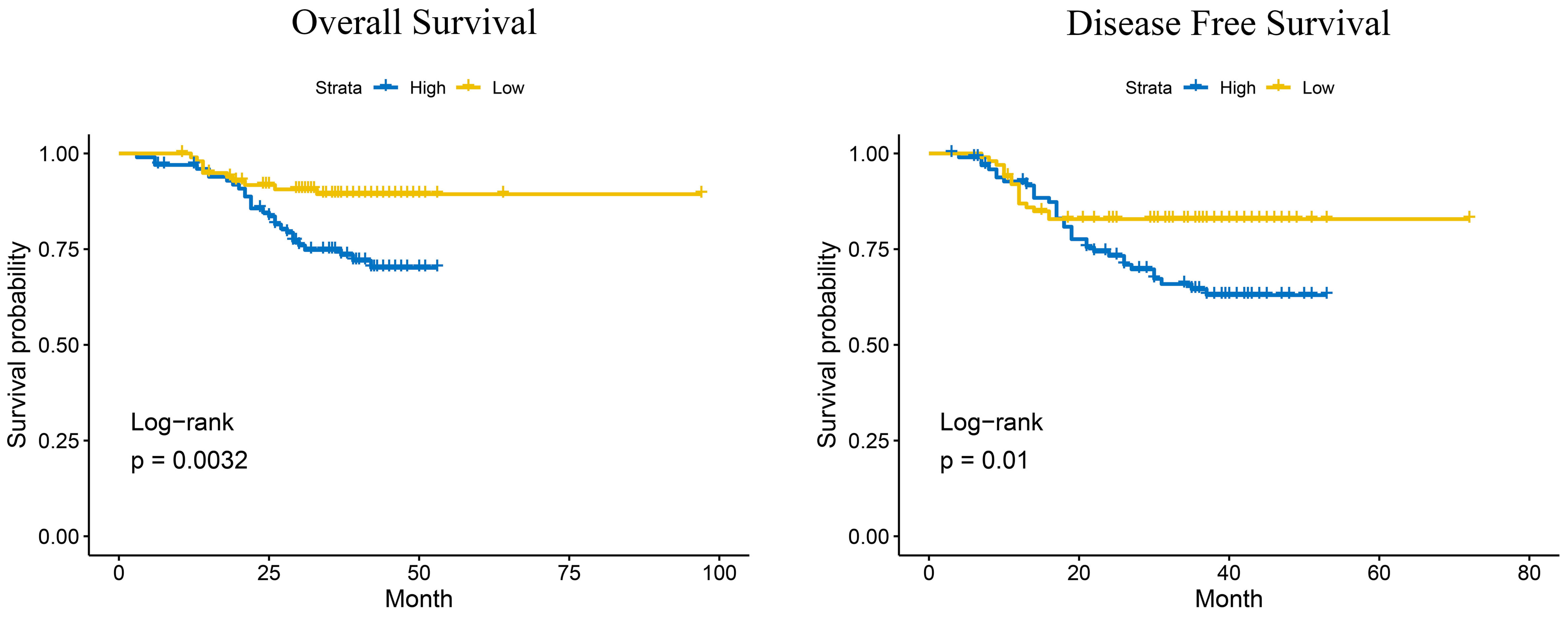

Overall Survival
Strata High Low
Survival probability
1.00
0.75
0.50
0.25
0.00
Log−rank
p = 0.0032
0
25
50
75
100
Month
Disease Free Survival
Strata High Low
Survival probability
1.00
0.75
0.50
0.25
0.00
Log−rank
p = 0.01
0
20
40
60
80
Month

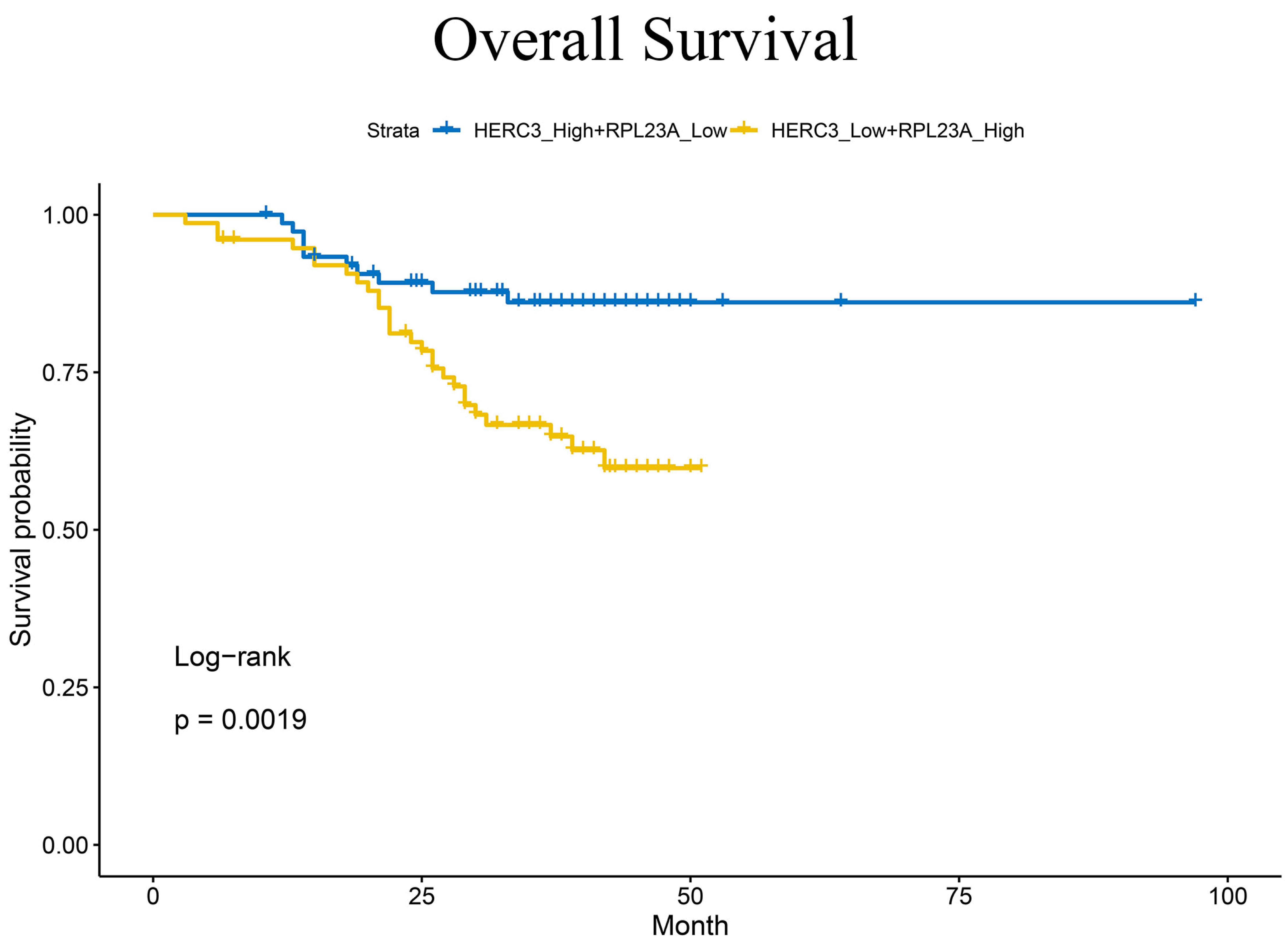

Overall Survival
Strata HERC3_High+RPL23A_Low HERC3_Low+RPL23A_High
Survival probability
1.00
0.75
0.50
0.25
0.00
Log−rank
p = 0.0019
0
25
50
75
100
Month

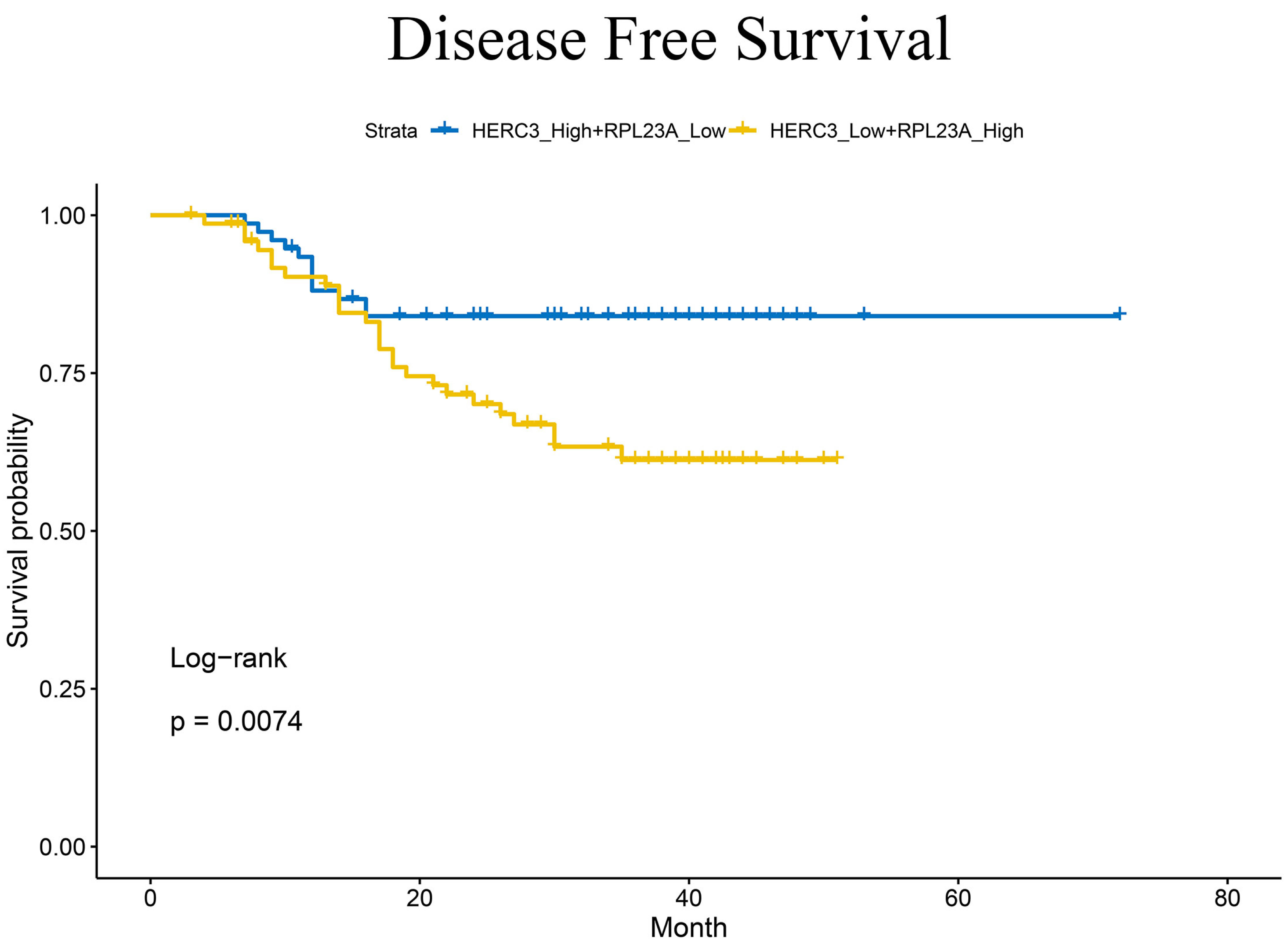

Disease Free Survival
Strata HERC3_High+RPL23A_Low HERC3_Low+RPL23A_High
Survival probability
1.00
0.75
0.50
0.25
0.00
Log−rank
p = 0.0074
0
20
40
60
80
Month

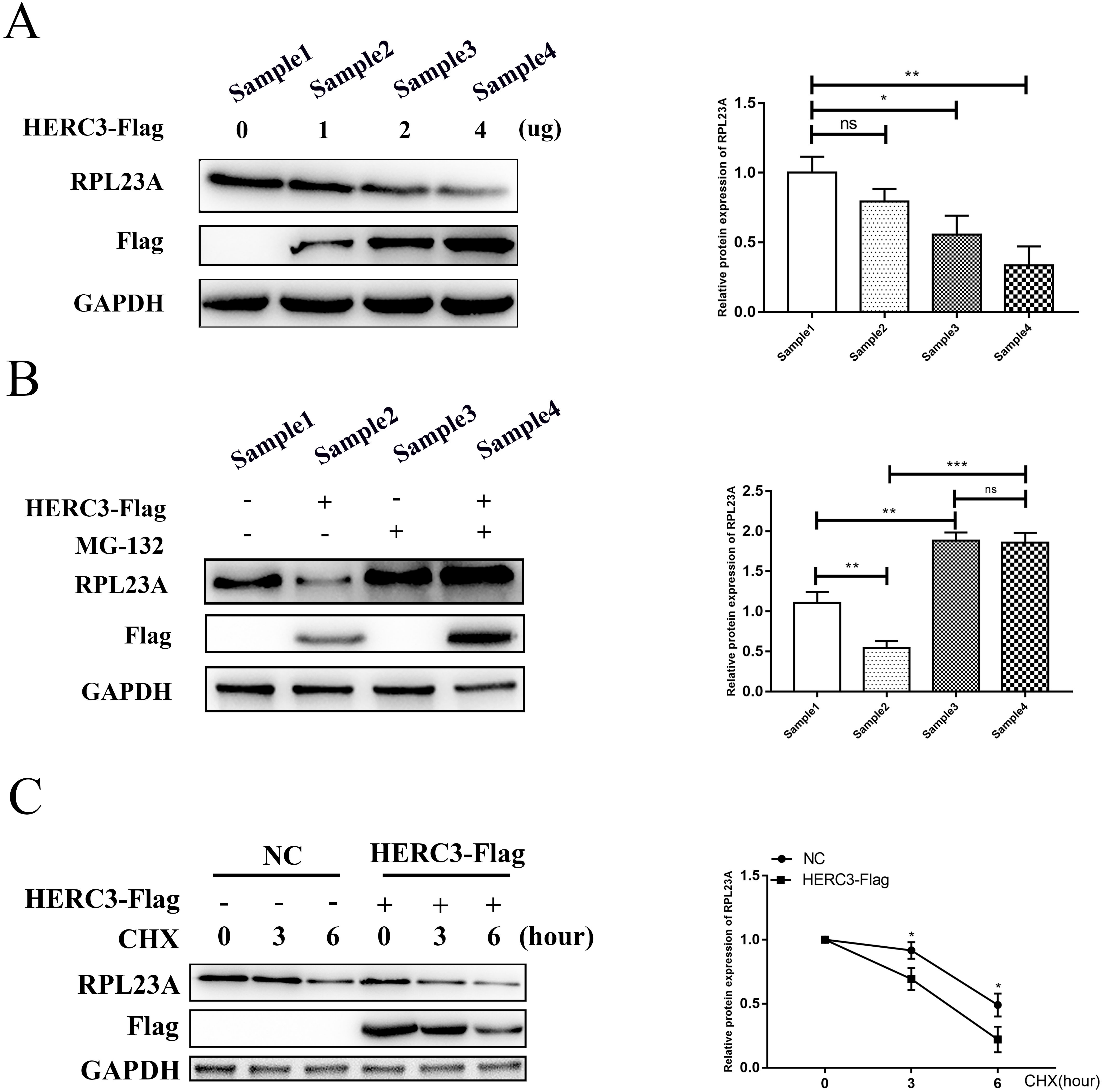
A
Sample1
Sample2
Sample3
Sample4
HERC3-Flag 0 1 2 4 (ug)
RPL23A
Flag
GAPDH
Relative protein expression of RPL23A
ns
*
**
B
Sample1
Sample2
Sample3
Sample4
HERC3-Flag - + - +
MG-132 - - + +
RPL23A
Flag
GAPDH
Relative protein expression of RPL23A
**
***
ns
C
NC
HERC3-Flag
HERC3-Flag - - - + + +
CHX 0 3 6 0 3 6 (hour)
RPL23A
Flag
GAPDH
NC
HERC3-Flag
Relative protein expression of RPL23A
CHX(hour)

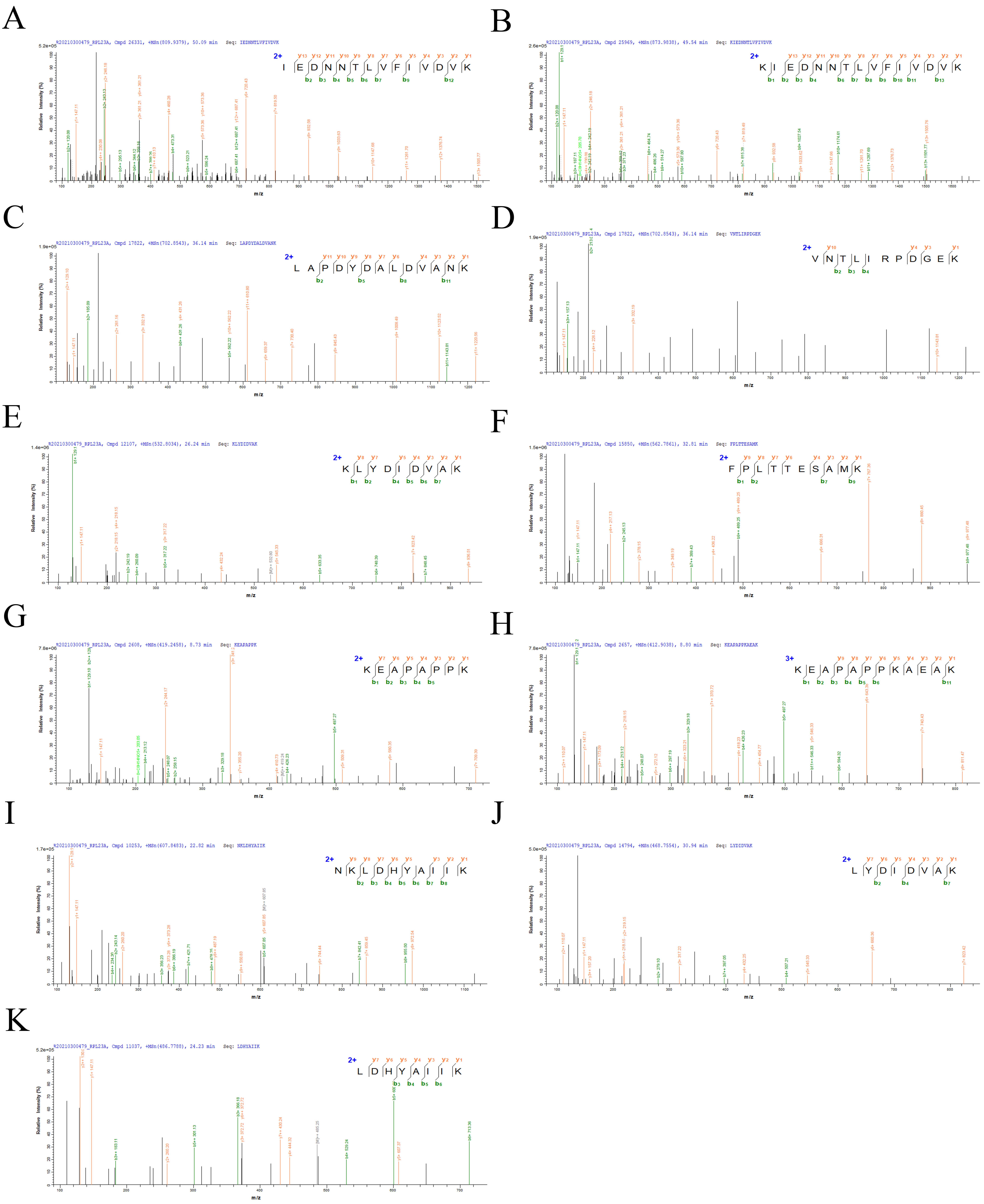
A
R20210300479_RPL23A, Cmpd 26331, +MSn(809.9379), 50.09 min Seq: IEDNNTLVFIVDVK
B
R20210300479_RPL23A, Cmpd 25969, +MSn(873.9838), 49.54 min Seq: KIEDNNTLVFIVDVK
C
R20210300479_RPL23A, Cmpd 17822, +MSn(702.8543), 36.14 min Seq: LAPDYDALDVANK
D
R20210300479_RPL23A, Cmpd 17822, +MSn(702.8543), 36.14 min Seq: VNTLIRPDGEK
E
R20210300479_RPL23A, Cmpd 12107, +MSn(532.8034), 26.24 min Seq: KLYDIDVAK
F
R20210300479_RPL23A, Cmpd 15850, +MSn(562.7861), 32.81 min Seq: FPLTTESAMK
G
R20210300479_RPL23A, Cmpd 2608, +MSn(419.2458), 8.73 min Seq: KEAPAPPK
H
R20210300479_RPL23A, Cmpd 2657, +MSn(412.9038), 8.80 min Seq: KEAPAPPKAEAK
I
R20210300479_RPL23A, Cmpd 10253, +MSn(607.8483), 22.82 min Seq: NKLDHYAIIK
J
R20210300479_RPL23A, Cmpd 14794, +MSn(468.7554), 30.94 min Seq: LYDIDVAK
K
R20210300479_RPL23A, Cmpd 11037, +MSn(486.7788), 24.23 min Seq: LDHYAIIK
Relative Intensity (%)
m/z
